# Synthesis and biological evaluation of novel bi-gold mitocans in lung cancer cells

**DOI:** 10.3389/fchem.2023.1292115

**Published:** 2023-12-11

**Authors:** Wenwen Ding, Qingbin Cui, Wenhua Lu, Yongliang Du, Yao Luo, Yumin Hu, Peng Huang, Shijun Wen

**Affiliations:** State Key Laboratory of Oncology in South China, Collaborative Innovation Center for Cancer Medicine, Guangdong Provincial Clinical Research Center for Cancer, Department of Experimental Research, Sun Yat-sen University Cancer Center, Guangzhou, China

**Keywords:** mitochondria, apoptosis, reactive oxygen species, anticancer, bi-gold mitocans

## Abstract

Mitochondria are promising drug target for cancer treatment. We previously demonstrated that a bi-gold compound BGC2a was more potent than the mono-gold drug auranofin in suppressing cancer cells due to increased gold atom number that led to higher drug accumulation in and thereby inhibition of mitochondria. To exploit the potential of this new strategy, we further designed and synthesized a series of bi-gold mitocans, the compounds targeting mitochondria. The results showed that most of the newly synthesized mitocans exhibited obviously lower IC_50_ than auranofin, an old drug that is repurposed in clinical trials for cancer treatment. The best mitocan C3P4 was nearly 2-fold more potent than BGC2a in human non-small cell lung cancer A549 cells and mantle cell lymphoma Jeko-1 cells, exhibiting substantial colony formation-suppressing and tumor-suppressing effects in A549 cells xenograft model. C3P4 induced apoptosis in a dose-dependent manner and arrested cell cycle at G0/G1 phase. The mechanistic study showed that C3P4 significantly increased the global reactive oxygen species and mitochondrial superoxide level, and reduced the mitochondrial membrane potential. C3P4 preferentially accumulated in mitochondria as measured by the gold content and substantially inhibited oxygen consumption rate and ATP production. These results further validated our hypothesis that targeting mitochondria would be promising to develop more potent anticancer agents. C3P4 may be further evaluated as a drug candidate for lung cancer treatment.

## 1 Introduction

Growing evidence has suggested that mitochondrion is a feasible target for cancer treatment (Cui et al., 2017; Murphy and Hartley, 2018; Wang et al., 2018). Mitochondrion plays fundamental roles in regulating many aspects of cell biology, including generating adenosine triphosphate (ATP), providing essential intermediates for constructing lipids, proteins and other biomolecules, maintaining redox homeostasis, and guarding cell death, etc. (Murphy and Hartley, 2018; Supinski et al., 2019). Functioning as the “powerhouse” of cells, mitochondria generate ATP through oxidative phosphorylation via the electron transport chain (ETC) on the inner membrane (Sousa et al., 2018). Another pivotal role that mitochondria lead is initializing and prompting the cascade of apoptosis procedure (Abate et al., 2019) via regulating key proteins in the apoptosis pathway (Tait and Green, 2010; Martinou and Youle, 2011; Bock and Tait, 2020). Furthermore, mitochondria are also the primary source of cellular reactive oxygen species (ROS), which may result from the reaction of oxygen with those escaped electrons from ETC in mitochondria (Murphy, 2009; Tirichen et al., 2021). Generally, ROS at normal level regulate cellular redox homeostasis and participate in signaling pathways that affect many cellular processes, thereby promoting cancer cells proliferation, survival, metastasis and modulating anticancer drug resistance (Cui et al., 2018; Kumari et al., 2018; Cui et al., 2019). On the other hand, high oxidative stress due to over-produced ROS may trigger apoptosis, making it a feasible strategy to increase ROS via pharmacological treatment to kill cancer cells (Chaiswing et al., 2018; Mani et al., 2022). Collectively, mitochondria can be rendered as a promising target for cancer therapy, and the development of mitocans, a term that is coined for those mitochondria-targeting anticancer agents, will be of importance (Ralph et al., 2006; Dong et al., 2020).

**FIGURE 1:**
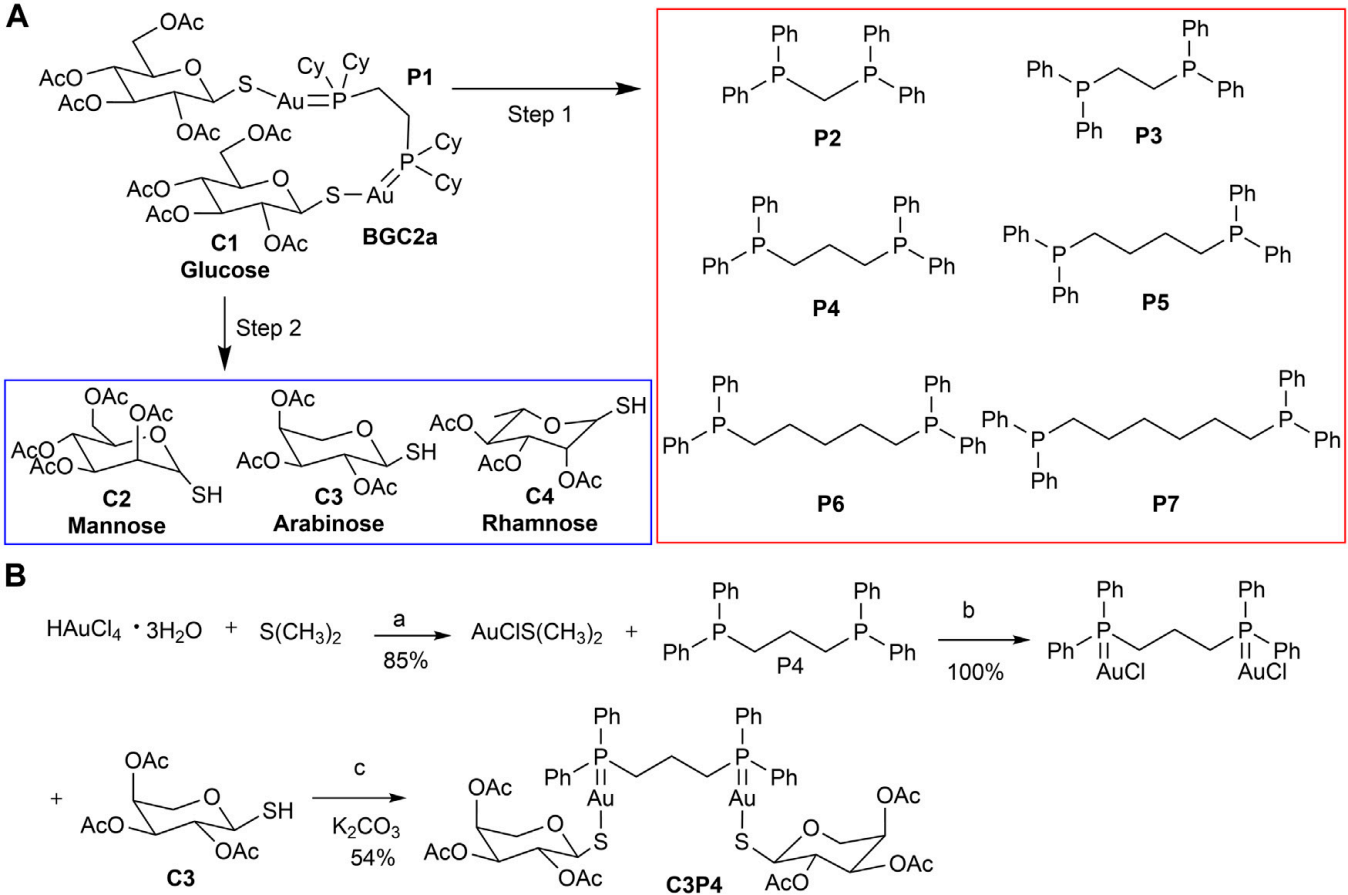
**(A)** The strategy of structural variation of mitocan BGC2a. The bi-phosphine ligand (P1) and two acetated glucose thiols (C1) in BGC2a were altered. The first step was to determine the most potent phosphine ligand by replacing P1 with other bi-phosphine ligands (P2-P7). The second step was to use the optimized phosphine ligand to coordinate with three other types of acetated monosaccharide thiols (C2-C4). **(B)** The synthesis of C3P4. Reagents and conditions: (a) EtOH, r.t.; (b) CH_2_Cl_2_, r.t.; (c) CH_2_Cl_2_/H_2_O, K_2_CO_3_, r.t.

Previously, we have demonstrated that a novel gold-based mitocan named BGC2a (Figure 1A), a bi-phosphine (1,2-bis(dicyclohexylphosphino) ethane (P1) coordinated with two gold atoms and linked with *O*-tetraacetylated 1-thiol-glucose (C1), preferentially accumulated in mitochondria of A549 lung cancer cells, inhibited oxygen consumption rate (OCR), and provoked remarkable ROS production globally, resulting in apoptosis and suppressing the tumor growth in both A549 and PANC-1 xenograft mice models (Cui et al., 2022). Given that mitochondria can be favorably targeted by positive-charged molecules (Cui et al., 2017; Wang et al., 2020), we hypothesized that the two gold atoms in BGC2a might play a central role in facilitating the mitochondria targeting and accumulating. In the current study, to further explore the anticancer potential of this type of bi-gold-based mitocans and to exploit more active agents for cancer treatment, we designed, synthesized and screened a series of bi-gold compounds coordinated with varied bi-phosphine ligands and different thiolated monosaccharides, and evaluated the anticancer activity of the most potent mitocan C3P4 *in-vitro* and *in-vivo*.

## 2 Materials and methods

### 2.1 Chemistry

Tetrachloroauric (III) acid (reagent grade) were purchased from HWRK Chem (Beijing, China), phosphine ligands and dimethyl sulfide were purchased from Alfa Aesar (Shanghai, China). Potassium carbonate and all solvents (analytical grade) used were purchased from Damao Chemical Reagent Factory (Tianjin, China). Column chromatography was generally performed on silica gel (200–300 mesh) and reactions were monitored by thin layer chromatography (TLC) on a glass pate coated with silica gel with fluorescent indicator (GF254) using UV light.

The gold-containing compounds were synthesized according to a reported procedure (Mirabelli et al., 1986; Lima and Rodriguez, 2011), and the synthetic route and details conditions of C3P4 were shown as an example in Figure 1B. Briefly, chloro(dimethylsulfide)gold(I) was prepared via the reduction of tetrachloroauric (III) acid (200 mg, 589 μmol) by dimethyl sulfide (146 mg, 2.35 mmol) in ethanol (room temperature, 4 h, 85% yield). The freshly prepared chloro (dimethylsulfide)gold(I) (147 mg, 499 μmol) reacted with bi-phosphine P4 (206 mg, 499 μmol) in dichloromethane to give the intermediate gold(I)-phosphine complex (room temperature, 2 h), which then coupled with C3 (146 mg, 499 μmol) under potassium carbonate (room temperature, 2–4 h) to give the target mitocan C3P4 (white solid, 374 mg, 54% yield). The other eight compounds were synthesized via the same method. In total, nine mitocans were synthesized in this study. ^1^H-NMR, ^13^C-NMR and and ^31^P-NMR spectra were recorded on a 400 MHz spectrometer (AV-400 Bruker) in CDCl_3_. Chemical shifts are given in ppm (δ) referenced to CDCl_3_ with 7.26 for ^1^H and 77.16 for ^13^C (Gandin et al., 2010; Cui et al., 2022). The original spectrum can be found in the Supporting materials.

**C1P2** (45%): ^1^H NMR (400 MHz, CDCl_3_) δ 7.71–7.59 (m, 8H), 7.45–7.29 (m, 12H), 5.27–5.20 (m, 2H), 5.16 (t, *J* = 9.3 Hz, 2H), 5.00 (td, *J* = 9.6, 2.0 Hz, 4H), 4.11 (dd, *J* = 12.2, 4.4 Hz, 2H), 4.03 (dd, *J* = 12.1, 2.4 Hz, 2H), 3.84–3.78 (m, 2H), 3.73 (t, *J* = 11.4 Hz, 2H), 2.04 (s, 6H), 1.99 (s, 6H), 1.97 (s, 6H), 1.83 (s, 6H). ^13^C NMR (101 MHz, CDCl_3_) δ 170.48, 170.34, 169.95, 143.28, 143.15, 133.81, 133.72, 132.49, 132.40, 131.84, 126.90, 126.81, 126.47, 125.92, 80.50, 77.55, 77.23, 76.91, 75.94, 72.63, 69.47, 65.93, 23.64, 23.53, 21.34, 21.11, 20.98, 17.74. ^31^P NMR (162 MHz, CDCl_3_) δ 30.85.

**C1P3** (63%): a known compound. ^1^H NMR (400 MHz, CDCl_3_) δ 7.80–7.59 (m, 8H), 7.53–7.32 (m, 12H), 5.23–4.94 (m, 8H), 4.15 (dd, *J* = 12.3, 4.9 Hz, 2H), 4.04 (dd, *J* = 12.3, 2.4 Hz, 2H), 3.76–3.64 (m, 2H), 2.70 (s, 4H), 1.98 (s, 6H), 1.96 (s, 6H), 1.89 (s, 6H), 1.82 (s, 6H).

**C1P4** (58%): ^1^H NMR (400 MHz, CDCl_3_) δ 7.75–7.65 (m, 8H), 7.48–7.46 (m, 12H), 5.10 (m, 8H), 4.17 (dd, *J* = 12.2, 4.3 Hz, 2H), 4.06 (d, *J* = 12.2 Hz, 2H), 3.73 (d, *J* = 7.7 Hz, 2H), 2.83 (d, *J* = 3.1 Hz, 4H), 2.00 (s, 6H), 1.99 (s, 6H), 1.96 (s, 6H), 1.95–1.89 (m, 2H), 1.84 (s, 6H). ^13^C NMR (101 MHz, CDCl_3_) δ 170.90, 170.44, 169.85, 169.80, 133.75, 133.68, 133.62, 133.55, 132.02, 130.08, 129.63, 129.60, 129.52, 129.49, 83.36, 77.80, 75.87, 74.45, 69.16, 63.00, 28.76, 28.65, 28.42, 28.31, 21.28, 20.89, 20.84, 20.41. ^31^P NMR (162 MHz, CDCl_3_) δ 32.19.

**C1P5** (63%): ^1^H NMR (400 MHz, CDCl_3_) δ 7.76–7.61 (m, 8H), 7.53–7.39 (m, 12H), 5.20–4.97 (m, 8H), 4.19 (dd, *J* = 12.2, 4.9 Hz, 2H), 4.08 (dd, *J* = 12.2, 2.4 Hz, 2H), 3.74 (ddd, *J* = 9.7, 4.8, 2.4 Hz, 2H), 2.47–2.34 (m, 4H), 2.03 (s, 6H), 1.99 (s, 6H), 1.93 (s, 6H), 1.89 (s, 6H), 1.61 (s, 4H), 1.47 (m, 4H). ^13^C NMR (101 MHz, CDCl_3_) δ 170.90, 170.43, 169.79, 169.77, 133.59, 133.46, 133.35, 131.80, 130.99, 130.75, 130.21, 129.44, 129.40, 129.33, 129.29, 83.34, 77.85, 75.91, 74.44, 69.22, 63.11, 30.54, 30.38, 28.20, 27.86, 25.60, 21.30, 20.90, 20.87, 20.83. ^31^P NMR (162 MHz, CDCl_3_) δ 34.83.

**C1P6** (52%): ^1^H NMR (400 MHz, CDCl_3_) δ 7.77–7.62 (m, 8H), 7.55–7.38 (m, 12H), 5.20–4.97 (m, 8H), 4.18 (dt, *J* = 11.9, 6.0 Hz, 2H), 4.12–4.03 (m, 2H), 3.74 (ddd, *J* = 9.7, 4.8, 2.5 Hz, 2H), 2.40 (d, *J* = 9.9 Hz, 4H), 2.00 (s, 12H), 1.94 (s, 6H), 1.88 (s, 6H), 1.65 (s, 8H). ^13^C NMR (101 MHz, CDCl_3_) δ 170.72, 170.25, 169.60, 133.44, 133.33, 133.31, 133.20, 131.68, 130.60, 130.06, 129.33, 129.30, 129.21, 129.19, 83.15, 77.66, 75.74, 74.25, 69.02, 62.89, 27.68, 27.34, 24.60, 21.10, 20.72, 20.69, 20.64. ^31^P NMR (162 MHz, CDCl_3_) δ 34.70.

**C1P7** (63%): ^1^H NMR (400 MHz, CDCl_3_) δ 7.76–7.60 (m, 8H), 7.51–7.39 (m, 12H), 5.21–4.96 (m, 8H), 4.26–4.15 (m, 2H), 4.13–4.04 (m, 2H), 3.79–3.68 (m, 2H), 2.46 (d, *J* = 8.4 Hz, 4H), 2.03 (s, 6H), 2.00 (s, 6H), 1.90 (s, 6H), 1.88 (s, 6H), 1.84–1.63 (m, 8H). ^13^C NMR (101 MHz, CDCl_3_) δ 170.69, 170.19, 169.63, 169.59, 133.39, 133.28, 133.26, 133.15, 131.73, 130.44, 130.36, 129.90, 129.82, 129.32, 129.29, 129.21, 129.18, 83.17, 77.78, 75.74, 74.25, 69.01, 62.93, 27.89, 27.55, 27.02, 26.97, 26.84, 26.80, 21.14, 20.73, 20.65. ^31^P NMR (162 MHz, CDCl_3_) δ 34.94.

**C2P4** (73%): ^1^H NMR (400 MHz, CDCl_3_) δ 7.69–7.65 (m, 8H), 7.46 (s, 12H), 5.95 (d, *J* = 10.0 Hz, 2H), 5.84 (s, 2H), 5.50 (s, 2H), 5.33 (t, *J* = 10.0 Hz, 2H), 4.78 (d, *J* = 9.9 Hz, 2H), 4.37–4.24 (m, 2H), 4.03 (d, *J* = 12.3 Hz, 2H), 2.99–2.73 (m, 4H), 2.13 (s, 6H), 2.02 (s, 12H), 1.92 (m, 8H). ^13^C NMR (101 MHz, CDCl_3_) δ 171.06, 170.39, 170.04, 169.97, 133.78, 133.65, 133.60, 133.47, 132.08, 132.00, 129.97, 129.65, 129.61, 129.54, 129.50, 129.17, 80.77, 77.55, 77.23, 76.91, 76.00, 69.59, 68.41, 67.16, 62.95, 28.72, 28.61, 28.38, 28.27, 21.31, 20.98, 20.95, 20.26. ^31^P NMR (162 MHz, CDCl_3_) δ 31.85.

**C3P4** (54%): ^1^H NMR (400 MHz, CDCl_3_) δ 7.81–7.63 (m, 8H), 7.43 (s, 12H), 5.26 (t, *J* = 9.3 Hz, 2H), 5.21 (s, 2H), 5.08 (d, *J* = 8.9 Hz, 2H), 4.96 (d, *J* = 9.7 Hz, 22H), 3.98 (d, *J* = 13.1 Hz, 2H), 3.63 (d, *J* = 13.1 Hz, 2H), 2.79 (dd, *J* = 15.6, 7.4 Hz, 4H), 2.00 (s, 6H), 1.97 (s, 6H), 1.95–1.86 (m, 2H), 1.74 (s, 6H). ^13^C NMR (101 MHz, CDCl_3_) δ 170.51, 170.32, 169.96, 133.69, 133.55, 131.89, 130.28, 130.14, 129.74, 129.60, 129.49, 129.38, 83.78, 75.20, 71.87, 69.21, 67.61, 28.86, 28.74, 28.53, 28.41, 21.31, 20.89, 20.82, 20.37. ^31^P NMR (162 MHz, CDCl_3_) δ 32.06.

**C4P4** (65%): ^1^H NMR (400 MHz, CDCl_3_) δ 7.70–7.65 (m, 8H), 7.46 (s, 12H), 5.92 (d, *J* = 10.1 Hz, 2H), 5.77 (s, 2H), 5.51 (s, 2H), 5.07 (t, *J* = 9.8 Hz, 2H), 4.70–4.52 (m, 2H), 2.98–2.73 (m, 4H), 2.18–1.84 (m, 20H), 1.18 (d, *J* = 6.0 Hz, 6H). ^13^C NMR (101 MHz, CDCl_3_) δ 170.29, 170.22, 169.87, 133.59, 133.46, 133.32, 131.81, 131.74, 129.88, 129.67, 129.40, 129.37, 129.33, 129.29, 129.26, 129.12, 80.04, 76.11, 72.23, 69.32, 66.23, 28.54, 28.42, 28.20, 28.09, 21.17, 20.93, 20.83, 20.16. ^31^P NMR (162 MHz, CDCl_3_) δ 32.20.

### 2.2 Cells and cell cultures

Human non-small cell lung carcinoma cell line A549, and human mantle cell lymphoma cell line Jeko-1 were purchased from the American Type Culture Collection (ATCC, Rockville, MD, United States). Cells were cultured in RPMI 1640 medium (Gibco-BRL, Paisley, United Kingdom) supplemented with 10% or 20% fetal bovine serum (FBS, Gibco-BRL, Paisley, United Kingdom). All cells were incubated in a humidified incubator at 37 °C supplemented with 5% of carbon dioxide (CO_2_).

### 2.3 Cell viability

A549 cells were seeded in 96-well plate at a density of 3 × 10^3^/well and incubated (37 °C, 5% CO_2_) overnight. Jeko-1 cells were seeded at a density of 3 × 10^4^/well without further incubation. All nine mitocans, C1P2-C1P7, C2P4, C3P4 and C4P4, or control auranofin were dissolved in DMSO and then diluted with culture medium to different concentrations (0.01, 0.03, 0.1, 0.3, 1, 3, 6 μM) and then added to each well and co-incubated for 72 h (of note, the maximum concentration of DMSO in medium was 0.06%). At the end of treatment, 20 μL of MTS reagent was added to each well and incubated for another 3 h at 37°C. Absorbance was measured under 490 nm. The IC_50_ (concentration of a drug causing 50% inhibition of cell growth, and the data were repeated at least three times) value of each compound was calculated by GraphPad Prism 8.4 software (San Diego, CA, United States).

### 2.4 Apoptosis assay

A549 cells were seeded in 6-well plate at a density of 3–5 × 10^5^ cells/well and incubated in a humidified atmosphere (37°C, 5% CO_2_) over night. Then they were pretreated with C3P4 (2.5, 5 and 7.5 μM) for 48 h. Cells were collected, washed with PBS and stained with Annexin-V-FITC and PI (KeyGEN, Nanjing, China) for analysis of cell apoptosis using flow cytometer (Gallios, Beckman Coulter, Brea, CA, United States).

### 2.5 Cell cycle assay

A549 cells were seeded in 6-well plate at a density of 3–5 × 10^5^ cells/well and incubated in a humidified atmosphere (37°C, 5% CO_2_) over night. Then they were pretreated with C3P4 (0.1, 0.3 and 1 μM) for 24 h. Cells were collected, washed once with 1 mL PBS and then resuspended with 500 μL of cold 70% EtOH/H_2_O solution for 4 h, After removal of ethanol through centrifugation, washed with PBS and stained with PI/RNase (BD Biosciences, NJ, United States) for analysis using flow cytometer (Gallios, Beckman Coulter, Brea, CA, United States).

### 2.6 Colony formation assay

A549 cells in 2 mL RPMI 1640 medium with 10% FBS were seeded in a 6-well plate at a density of 400/well and incubated in a humidified atmosphere (37°C, 5% CO_2_) overnight. Then the cells were treated with C3P4 (0.1, 0.3, and 1 μM) for 14 days. The medium was removed and the cells were washed with PBS and incubated with 1 mL MeOH for 30 min. Then, 1 mL crystal violet (Crystal Violet Staining Solution, Beyotime, Shanghai, China) was added to each well, incubated in room temperature for 30 min, and gently washed by running water. After dryout, the colony number was counted by gel imaging and analysis system (ProteinSimple, Alphalmager HP, Santa Clara, CA, United States).

### 2.7 Cellular TrxR activity

A549 cells were seeded in 6-well plate of 3–5 × 10^5^/well, incubated in a humidified atmosphere (37°C, 5% CO_2_) overnight, and then 0.3 and 1 μM auranofin or C3P4 were added into each well and incubated for another 4 h. Thioredoxin Reductase Activity Colorimetric Assay Kit (Cat No. K763-100, Abcam) was applied to examine the cellular enzyme activity according to the manufacturer specification.

### 2.8 Mitochondria membrane potential (MMP), ROS levels, superoxide and mitochondrial mass

After treated with C3P4 for 2 or 6 h, A549 cells were collected and stained with Rhodamine-123 (1 μM, 20 min), or DC-FDA (1 μM, 40 min), or MitoSOX™ Red (5 μM, 20 min), or Mito Tracker green (0.3 μM, 20 min), and kept in the dark at 37°C with 5% CO_2_. The cells were then washed twice and re-suspended with PBS, followed by flow cytometry analysis using FACS Calibur flow cytometer (BD Biosciences, San Diego, CA, United States).

### 2.9 Cellular oxygen consumption assay (OCR)

OCR was measured by the Seahorse Bioscience Extracellular Flux Analyzer (XF24, Seahorse Bioscience, Billerica, MA, United States) in accordance with manufacturer’s instructions and our previous methods (Cui et al., 2022). The XF Assay Media was supplemented with glucose (5.5 mM) and pyruvate (1 mM) and the pH was adjusted to 7.4. OCR was recorded approximately every 8 min under basal condition. Upon C3P4 (1 and 3 μM), added a saturating concentration of oligomycin (1 μM), FCCP (1 μM) and Rotenone (0.5 μM). Coupled respiration was expressed as the percent decrease from basal respiration. Maximal FCCP respiration was taken as the highest measurement after addition of FCCP.

### 2.10 Gold content in mitochondria

A549 cells were seeded at dense of 4 × 10^6^/well in 25 cm^2^ cell culture flask and incubated in a humidified atmosphere (37 °C, 5% CO_2_) overnight. Then C3P4 or auranofin were added to make a final concentration of 0.3, 1 or 3 μM and pretreated for 2 h. Mitochondria were then collected according to the manufacturer specification of Qproteome Mitochondria Isolation kit (Cat No. 37612, Qiagen). The isolated mitochondria were treated with fuming HNO_3_ at 60 °C for 1 h and then were diluted with distilled water. Gold content was evaluated by inductively coupled plasma mass spectrometry (ICP-MS, iCAP Q, Thermo Scientific) as previously reported (Cui et al., 2022). The gold content in whole cells was detected similarly, and the percentage of gold in mitochondria in different groups were calculated.

### 2.11 *In-vivo* tumor growth inhibition experiments

Animal study was conducted following an approved protocol by Institutional Animals Care and Use Committee of Sun Yat-sen University Cancer (L102012016060L). Immune-deficient BALB/c nude mice (6-week-old, six to seven mice/group) were purchased from Vital River (Beijing, China), and cared for according to the guidelines of the Laboratory Animal Unit of Sun Yat-sen University. After adaptive 1 week, 4 × 10^6^ A549 cells were subcutaneously injected into the back flanks. After the tumor volumes reached 30–50 mm^3^, the mice were randomly divided into three groups, vehicle control group, 3 mg/kg C3P4 and 10 mg/kg auranofin via intraperitoneal (ip) or intravenous (iv) injection. C3P4 was delivered in a designed soluble system (96% PBS, 2% DMSO, 2% Solutol HS-15). The mice were treated 3 times a week on Monday, Wednesday and Friday. Mice weight and their tumor sizes were measured 2 times a week. After treatment, the mice were sacrificed via cervical dislocation.

### 2.12 Statistical analysis

Each experiment was repeated at least three times. The *in-vitro* data were presented as mean ± S.D. Comparisons between groups were performed using the Student’s t*-*test or one-way or two-way ANOVA provided in the Graphpad Prism 8.4 software (GraphPad, San Diego, CA, United States). A *p*-value of less than 0.05 is considered statistically significant.

## 3 Results

### 3.1 Drug design and synthesis of bi-gold mitocans

We adopted a stepwise strategy to optimize these bi-gold compounds. The first step was to determine the best bi-phosphine ligand. Previously in BGC2a, 1,2-bis(dicyclohexylphosphino)ethane (P1) was used (Cui et al., 2022). In this study, six other commercially available bi-phosphine ligands varying the linker length (from 1 to 6 carbons) were firstly utilized to coordinate with glucose-1-thiol C1, including bis(diphenylphosphaneyl)methane (P2), 1,2-bis(diphenylphosphaneyl)ethane (P3), 1,3-bis(diphenylphosphaneyl)propane (P4), 1,4-bis(diphenylphosphaneyl)butane (P5), 1,5-bis(diphenylphosphaneyl)pentane (P6), and 1,6-bis(diphenylphosphaneyl)hexane (P7) as shown in Figure 1A. Second, the most potent bi-phosphine ligand determined in the first step would be used to coordinate with O-acetylated 1-thiol mannose (C2), or arabinose (C3), or rhamnose (C4) as listed in Figure 1A. To this end, nine mitocans were synthesized and evaluated for their anticancer potentials.

### 3.2 *In-vitro* anticancer potential screening identified C3P4 as the most potent mitocan

**FIGURE 2:**
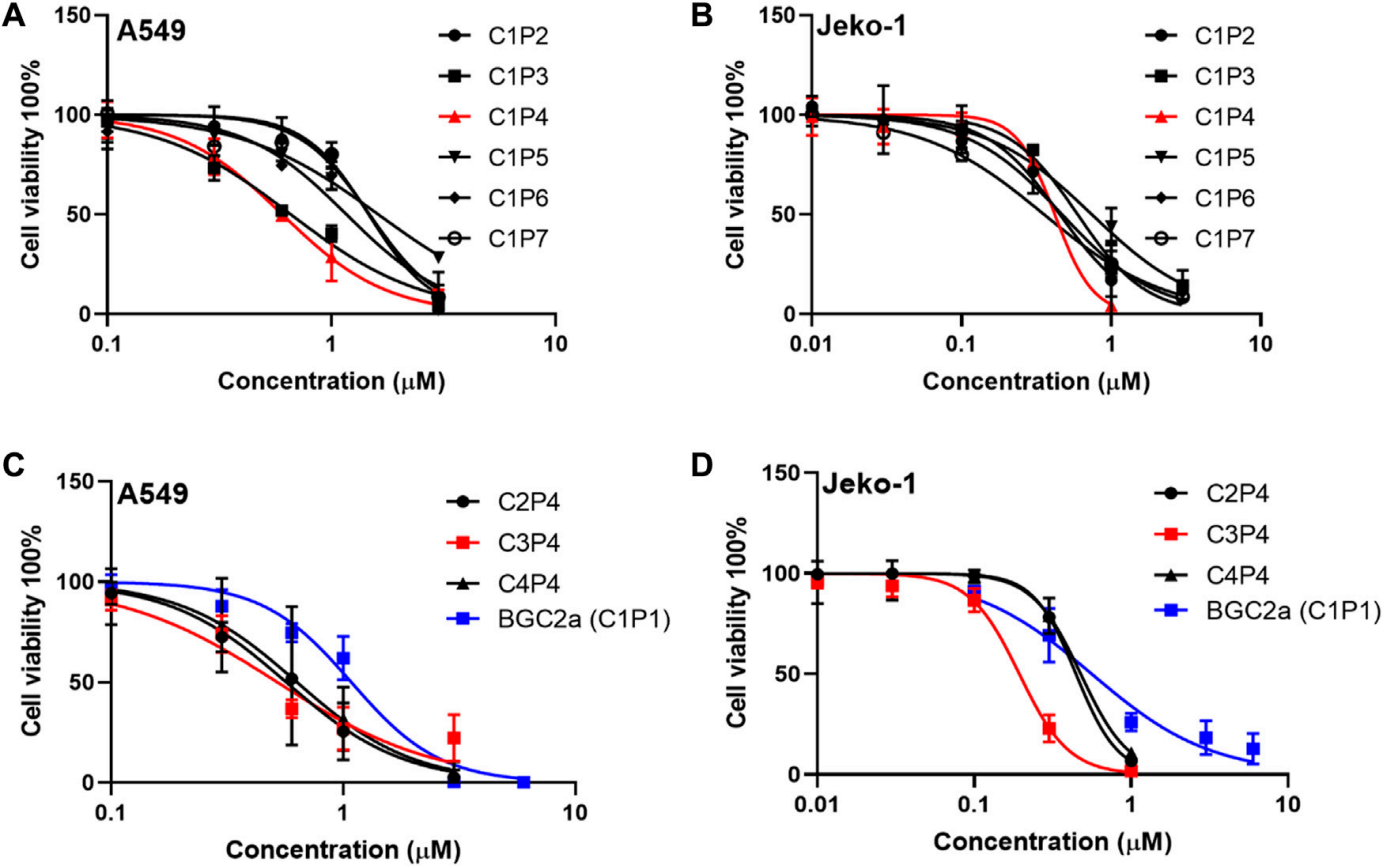
The effect of bi-gold compounds on viability of A549 and Jeko-1 cancer cells. **(A,B)** C1 series compounds in the first round. **(C,D)** P4 series compounds in the second round. BGC2a was included as a control.

The cytotoxicity of the first six compounds containing glucose C1 were tested in lung cancer A549 cells and validated in mantle cell lymphoma Jeko-1 cells. The results in Figure 2A; Table 1 showed that the most active mitocans were the one coordinated with phosphine ligand P4. It appeared that the optimum phosphine ligands were those with the linkers of 2- or 3-carbons in length. When the linker atom number reduced or increased, the anticancer activity decreased. The overall order of activity was C1P4 > C1P3 > C1P6 > C1P7 > C1P5 > C1P2 in C1 series compounds. It is also noticeable that all the six compounds were more potent than the known drug auranofin in A549 cells. The first six mitocans showed similar activities in Jeko-1 cells as shown in Figure 2B; Table 1.

**TABLE 1:** The IC_50_ values of bi-gold compounds in A549 and Jeko-1 cells.

Compound	IC_50_ (μM)^a^		Compound	IC_50_ (μM)	
	A549	Jeko-1		A549	Jeko-1
C1P2	1.45 ± 0.19	1.41 ± 0.17	C2P4	0.62 ± 0.04	0.31 ± 0.01
C1P3	0.66 ± 0.26	0.42 ± 0.13	C3P4	0.49 ± 0.06	0.21 ± 0.11
C1P4	0.61 ± 0.11	0.25 ± 0.06	C4P4	0.73 ± 0.30	0.36 ± 0.12
C1P5	1.50 ± 0.04	0.69 ± 0.19	Auranofin	5.56 ± 0.28	0.79 ± 0.08
C1P6	1.16 ± 0.08	0.37 ± 0.03	BGC2a (C1P1)	1.05 ± 0.01	0.42 ± 0.10
C1P7	1.47 ± 0.11	0.38 ± 0.01			

^a^ Represented by three independent experiments.

Since phosphine ligand P4 rendered the compound C1P4 the best, a second series of compounds C2P4, C3P4, C4P4 based on P4 were synthesized. Then, we tested these three P4 series compounds. The results in Figure 2C; Table 1 indicated that compound C3P4 was the best, and it has about 2-fold, or 10-fold lower IC_50_s in A549 cells than previously reported BGC2a or auranofin, respectively. Again, they showed the similar trend in Jeko-1 cells.

### 3.3 C3P4 showed great potential in suppressing the proliferation of A549 cells *in-vitro*

**FIGURE 3:**
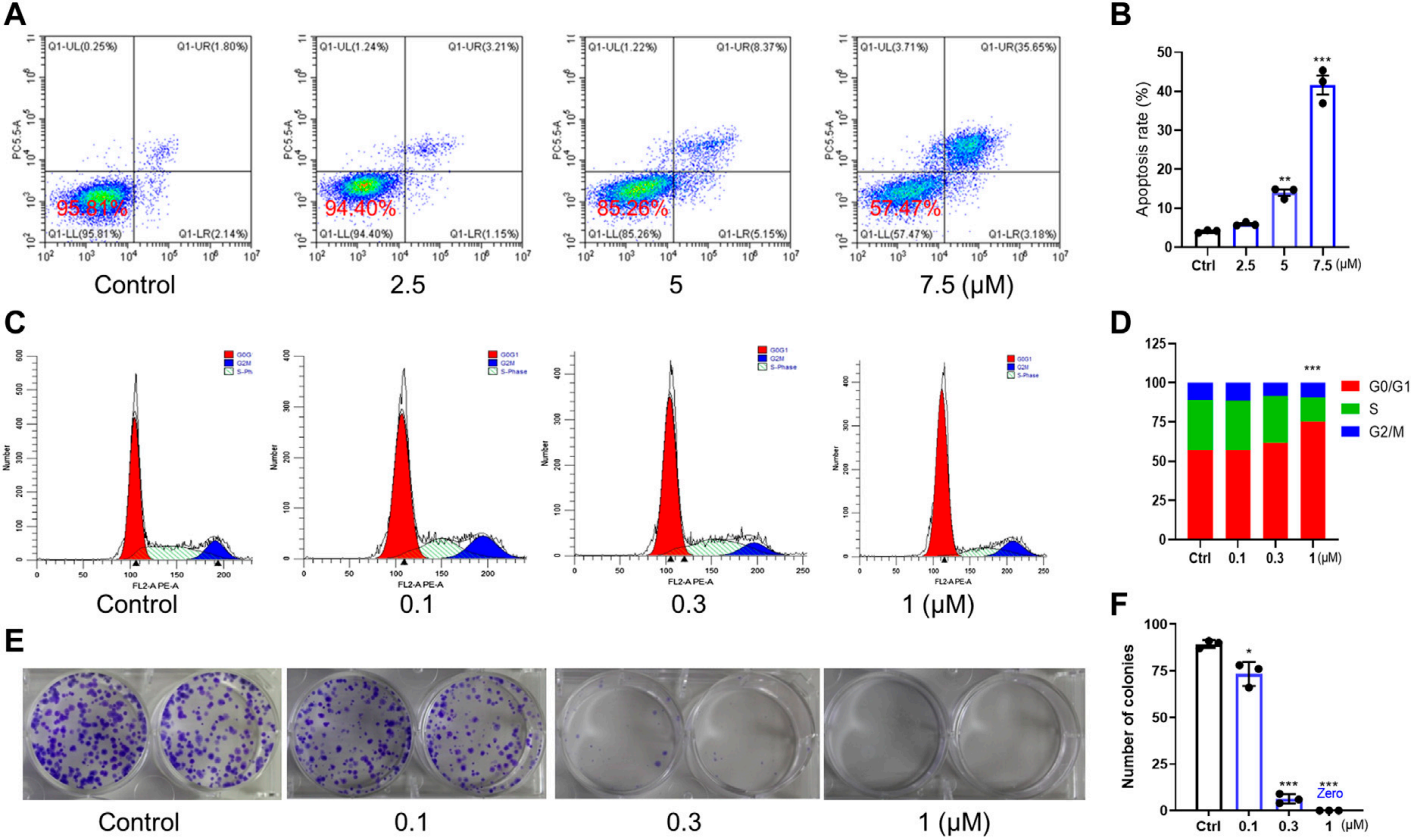
The effect of C3P4 on cell apoptosis and cell cycle. A549 cancer cells were used. **(A,B)** C3P4 induced apoptosis. **(C,D)** C3P4 caused cell cycle arrest at G0/G1 phase. **(E,F)** C3P4 dose-dependently suppressed colony formation. *, **, ***, *p* < 0.05, 0.01, 0.001 vs*.* control, respectively.

We further tested the anticancer potency of C3P4. The results in Figures 3A, B indicated that C3P4 could induce apoptosis of A549 in a concentration-dependent manner. Additionally, C3P4 could impact the cell cycle of A549 cells, with an arrest at G0/G1 phase as shown in Figures 3C, D, suggesting its potential in impacting DNA synthesis and mitosis. Furthermore, C3P4 could markedly suppress the colony formation of A549 cells at as low as 0.1 μM, and it can almost completely wipe out the colony at 1 μM (Figures 3E, F), which may suggest its potential ability to suppress cancer stem cells (Rajendran and Jain, 2018).

### 3.4 C3P4 impacted mitochondria’s functions

**FIGURE 4:**
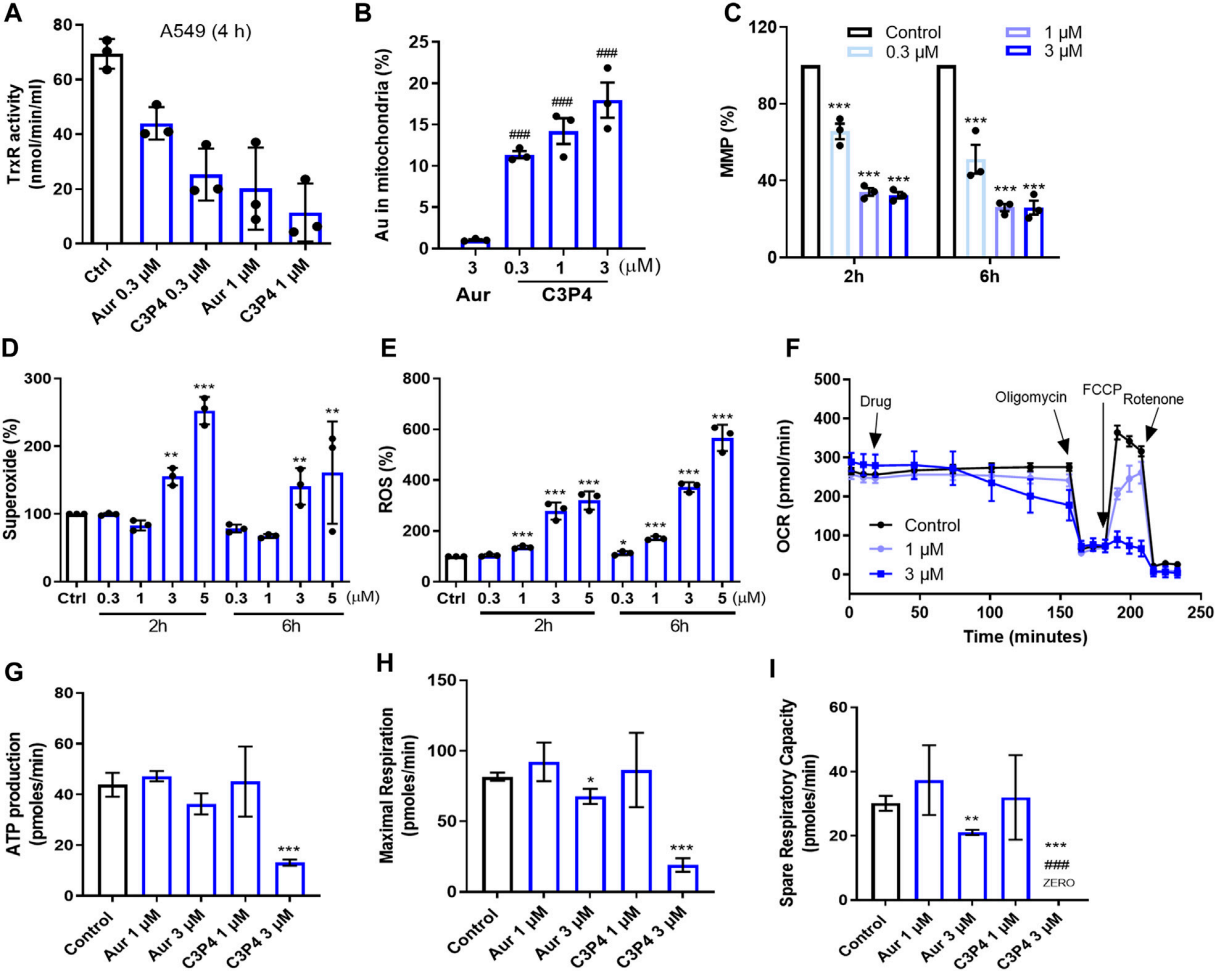
The effect of C3P4 on mitochondrial functions. **(A)** C3P4 dose-dependently inhibited cellular TrxR activity. **(B)** The intracellular gold ion accumulation in mitochondria after drug treatment. **(C–F)** C3P4 collapsed MMP **(C)**, increased mitochondrial superoxide **(D)** and global ROS **(E)**, and suppressed OCR **(F)**. **(G–I)** C3P4 reduced ATP production **(G)**, inhibited maximal respiration **(H)** and suppressed spare respiration capacity calculated from **(F)** ^###^, *p* < 0.001 vs*.* Auranofin; *, **, ***, *p* < 0.05, 0.01, 0.001 vs*.* Control. Aur stands for auranofin.

To determine the mechanism of action, we first tested if C3P4 could inhibit the cellular TrxR, the well-reported target of gold-based compounds (Pratesi et al., 2014; Garcia et al., 2016). The results in Figure 4A showed that C3P4 (0.3 and 1 μM) could dose-dependently inhibited TrxR in A549 cells after 4 hours treatment, more efficient than auranofin although with no statistically significant difference. Given that the ability of C3P4 to inhibit A549 cells was almost 10-fold higher active compared to auranofin, such potential of C3P4 could not be fully represented by its activity in suppressing TrxR, suggesting that TrxR inhibition alone might not be sufficient to effectively kill cancer cells and other critical players might be involved, consistent with our previous observation on BGC2a.

BGC2a was confirmed to enhance its mitochondrial accumulation and thereby impact the functions of mitochondria. We wanted to examine whether C3P4 possessed such effects on mitochondria. As shown in Figure 4B; Supplementary Figure S1, after 2 h treatment, there was significantly higher amount of mitochondrial gold (Au) content in C3P4 group at 0.3 μM than in auranofin group at 3 μM (Cui et al., 2022). These highly accumulated gold ions in mitochondria might contribute to dramatically decreased mitochondria membrane potential (MMP), and overproduced mitochondrial superoxide and global ROS in the whole cells (Figures 4C–E). The collapsed MMP and increased ROS levels may finally lead to mitochondria-mediated apoptosis, which needs further validation. C3P4 (3 and 5 μM, 2 h) slightly increased the mitochondria number (Supplementary Figure S2), indicating a possible compensatory mechanism in cancer cells to activate mitochondria biogenesis. Furthermore, C3P4 substantially inhibited OCR, ATP production, maximum and spare respiration (Figures 4F–I). These results further confirmed our hypothesis that gold compound might suppress the mitochondrial respiration, leading to the inhibition of cancer cells.

### 3.5 C3P4 inhibited tumor growth of A549 xenograft models

**FIGURE 5:**
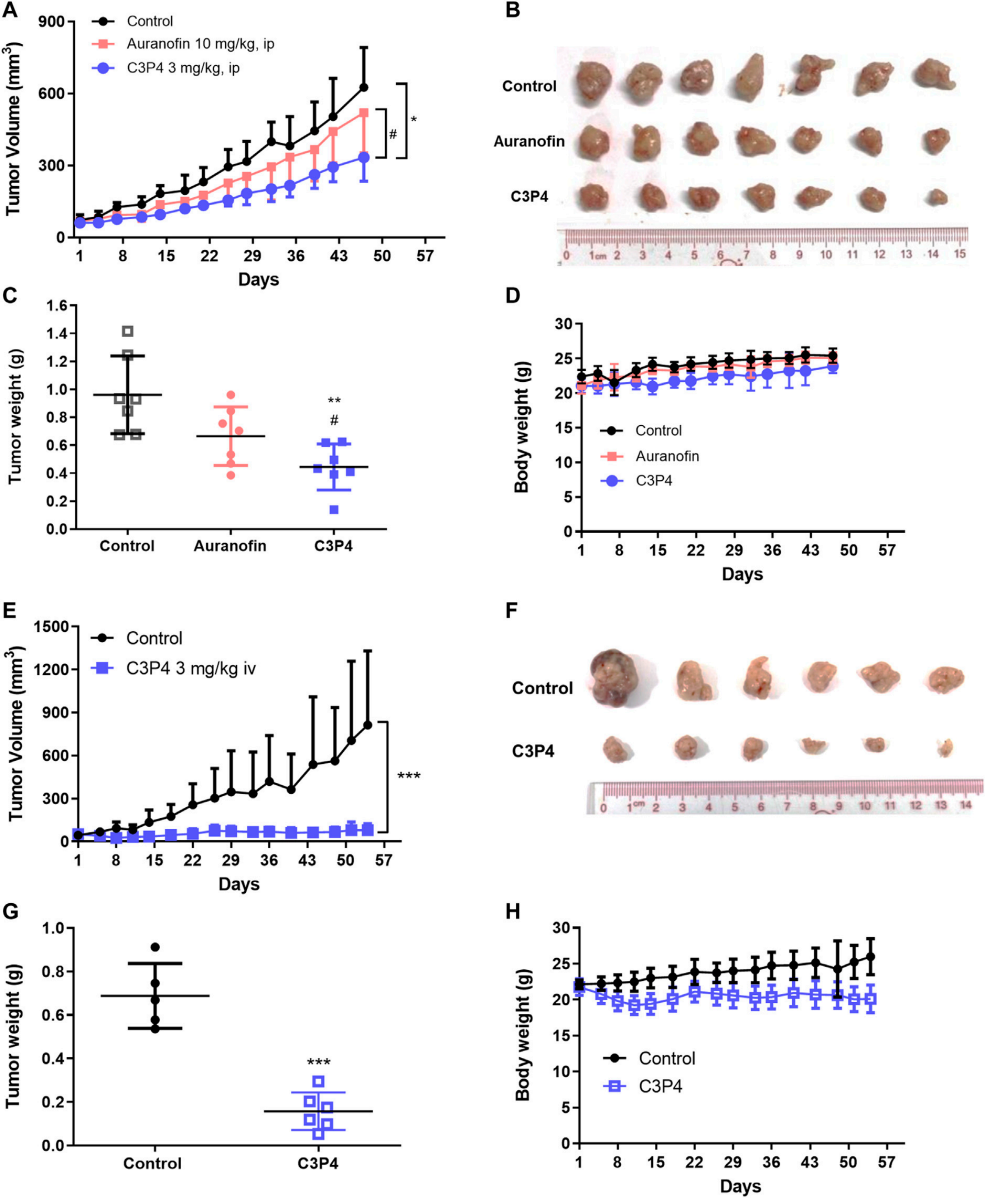
The *in vivo* antitumor growth efficacy of C3P4. A549 xenograft mice model was used. **(A–D)** The effect of C3P4 with ip injection showed great tumor-suppressing effects on tumor volumes **(A)**, tumor tissues **(B)**, tumor weight **(C)** and mice body weight **(D) (E–H)** The effect of C3P4 with iv injection on tumor volumes **(E)**, tumor tissues **(F)**, tumor weight **(G)** and mice body weight **(H)** *, **, ***, *p* < 0.05, 0.01 or 0.001, respectively, vs*.* the control group. ^#^ *p* < 0.05 vs. auranofin.

Finally, we determined C3P4’s tumor-suppressing effects in A549 xenograft models. Compared with the anti-tumor effect of auranofin treatment (10 mg/kg, ip), C3P4 administration (3 mg/kg, ip) significantly suppressed the tumor growth of A549 xenograft model (Figures 5A–C). Compound C3P4 did not affect mice weight as shown in Figure 5D. Additionally, when C3P4 was administered via iv injection at the same dose, the tumor growth was nearly completely inhibited with inhibition rate of 90% (Figures 5E–G). While the general behavior of the treated mice was normal, a slight weight loss was observed (Figure 5H). Further pharmacokinetic study of C3P4 is needed for the drug administration regimen optimization.

## 4 Discussion

Gold has been applied as medicine for many decades in East Asia (Kauffman, 1985; Yeo et al., 2018). Auranofin is a gold-based compound approved by FDA for rheumatoid arthritis in 1985. Growing evidence has suggested that the drug demonstrates therapeutic effects for infectious diseases (Capparelli et al., 2017; Tunes et al., 2020; Wallis et al., 2021) as well as for cancers (Steers et al., 2022; Szeliga and Rola, 2022). Clinical trials using auranofin for cancer therapy are undergoing (NCT03456700, ovarian cancer; NCT01737502, lung cancers). However, its approval for cancers remains pending despite of being tested in clinical trials for over 10 years (Halatsch et al., 2021). Thus, more effective gold-based anticancer agents are still in urgent need to combat cancers.

It is widely believed that auranofin works as a prodrug which converts to an active pharmacophore, gold ion, under physiological conditions to exert its pharmacological effects (Pratesi et al., 2014; Kim et al., 2023). Triethylphosphine gold ion might be the leading metabolite of auranofin to effectively target mitochondria and inhibit cancer cells. We previously validated that the increase of gold atom number in the structure of auranofin analogs, e.g., bi-gold mitocans BGC2a, could significantly enhance the cytotoxicity (Cui et al., 2022). In this study, we expanded the structural variations of bi-gold mitocans via coordinating various bi-phosphine ligands and different monosaccharide. The current study demonstrated that the newly synthesized bi-gold mitocans generally had higher cytotoxicity than the old drug auranofin in both A549 and Jeko-1 cells. Among all synthetic mitocans, C3P4 which had a 1,3-bis(diphenylphosphaneyl)propane ligand coordinated with arabinose was the best. The compound was about 10-fold and 4-fold more active than auranofin in A549 and Jeko-1 cells respectively and was 2-fold more active than BGC2a. More importantly, C3P4 (3 mg/kg) was more effective than auranofin (10 mg/kg) in suppressing tumor growth in A549 cells xenograft model. The preliminary mechanism study showed that C3P4 preferably accumulated in mitochondria and inhibited OCR, collapsed MMP, thereby resulted in apoptosis and cell cycle arrest of lung cancer A549 cells, inhibited colony formation and suppressed tumor growth, suggesting it as a promising new mitocan for lung cancer treatment.

The current study indicated that the different types of thiolated monosaccharides and phosphine ligands have dramatic influence on biological activity. Further modifications using other thiolated fragments and phosphine ligands are worth trying. Meanwhile, it remains unknown whether C3P4 could target and accumulate in tumor tissues as it did in cells. In addition, since C3P4’s major target remains unclear, an in-depth pharmacological study, for example, the associated signal pathways in mediating C3P4-induced apoptosis and cell cycle arrest, is necessary and currently under way.

## 5 Conclusion

In conclusion, our proof of concept of enhancing biological activity by increasing positive gold ions to target mitochondria may serve as a potential foundation for metal-based anticancer mitocans. After the structural variation of the previously developed potent bi-gold compound BGC2a, C3P4 is found to be the best to inhibit cancer cells and may serve as a drug candidate for further evaluations.

## Data Availability

The original data presented in the study are included in the article/Supplementary Material, and are available at http://www.researchdata.org.cn, further inquiries can be directed to the corresponding authors.

## References

[B1] Abate M. Festa A. Falco M. Lombardi A. Luce A. Grimaldi A. (2019). Mitochondria as playmakers of apoptosis, autophagy and senescence. Semin. Cell Dev. Biol. 98, 139–153. 10.1016/j.semcdb.2019.05.022 31154010

[B2] Bock F. J. Tait S. (2020). Mitochondria as multifaceted regulators of cell death. Nat. Rev. Mol. Cell Bio. 21, 85–100. 10.1038/s41580-019-0173-8 31636403

[B3] Capparelli E. V. Bricker-Ford R. Rogers M. J. McKerrow J. H. Reed S. L. (2017). Phase I clinical trial results of auranofin, a novel antiparasitic agent. Antimicrob. Agents Ch. 61, e01947 16. 10.1128/AAC.01947-16 PMC5192119 27821451

[B4] Chaiswing L. St C. W. St C. D. (2018). Redox paradox: a novel approach to therapeutics-resistant cancer. Antioxid. Redox Sign. 29, 1237–1272. 10.1089/ars.2017.7485 PMC6157438 29325444

[B5] Cui Q. Ding W. Liu P. Luo B. Yang J. Lu W. (2022), Developing Bi-gold compound BGC2a to target mitochondria for the elimination of cancer cells. Int. J. Mol. Sci. 23, 12169. 10.3390/ijms232012169 36293028 PMC9602679

[B7] Cui Q. Wang J. Q. Assaraf Y. G. Ren L. Gupta P. Wei L. (2018). Modulating ROS to overcome multidrug resistance in cancer. Drug resist. Update 41, 1–25. 10.1016/j.drup.2018.11.001 30471641

[B8] Cui Q. Wen S. Huang P. (2017). Targeting cancer cell mitochondria as a therapeutic approach: recent updates. Future Med. Chem. 9, 929–949. 10.4155/fmc-2017-0011 28636410

[B9] Cui Q. Yang Y. Ji N. Wang J. Q. Ren L. Yang D. H. (2019). Gaseous signaling molecules and their application in resistant cancer treatment: from invisible to visible. Future Med. Chem. 11, 323–336. 10.4155/fmc-2018-0403 30802141

[B10] Dong L. Gopalan V. Holland O. Neuzil J. (2020). Mitocans revisited: mitochondrial targeting as efficient anti-cancer therapy. Int. J. Mol. Sci. 21, 7941. 10.3390/ijms21217941 33114695 PMC7663685

[B11] Gandin V. Fernandes A. P. Rigobello M. P. Dani B. Sorrentino F. Tisato F. (2010). Cancer cell death induced by phosphine gold(I) compounds targeting thioredoxin reductase. Biochem. Pharmacol. 79, 90–101. 10.1016/j.bcp.2009.07.023 19665452

[B12] Garcia A. Machado R. C. Grazul R. M. Lopes M. T. Correa C. C. Dos S. H. (2016). Novel antitumor adamantane-azole gold(I) complexes as potential inhibitors of thioredoxin reductase. J. Biol. Inorg. Chem. 21, 275–292. 10.1007/s00775-016-1338-y 26841791

[B13] Halatsch M. E. Kast R. E. Karpel-Massler G. Mayer B. Zolk O. Schmitz B. (2021). A phase Ib/IIa trial of 9 repurposed drugs combined with temozolomide for the treatment of recurrent glioblastoma: CUSP9v3. Neurooncol Adv. 3, vdab075. 10.1093/noajnl/vdab075 34377985 PMC8349180

[B14] Kauffman G. B. (1985). The role of gold in alchemy. Part I. Gold Bull. 18, 31–44. 10.1007/BF03214684

[B15] Kim H. Y. Otgontenger U. Kim J. W. Lee Y. J. Kim S. B. Lim S. C. (2023). Anti-fibrotic effect of aurocyanide, the active metabolite of auranofin. Arch. Pharm. Res. 46, 149–159. 10.1007/s12272-023-01438-1 36894745 PMC9998255

[B16] Kumari S. Badana A. K. Malla R. Malla R. (2018). Reactive oxygen species: a key constituent in cancer survival. Biomark. Insights 13, 1177271918755391. 10.1177/1177271918755391 29449774 PMC5808965

[B17] Lima J. C. Rodriguez L. (2011). Phosphine-gold(I) compounds as anticancer agents: general description and mechanisms of action. Anti-Cancer Agent Me 11, 921–928. 10.2174/187152011797927670 21864238

[B18] Mani S. Swargiary G. Ralph S. J. (2022). Targeting the redox imbalance in mitochondria: a novel mode for cancer therapy. Mitochondrion 62, 50–73. 10.1016/j.mito.2021.11.002 34758363

[B19] Martinou J. C. Youle R. J. (2011). Mitochondria in apoptosis: bcl-2 family members and mitochondrial dynamics. Dev. Cell 21, 92–101. 10.1016/j.devcel.2011.06.017 21763611 PMC3156409

[B20] Mirabelli C. K. Johnson R. K. Hill D. T. Faucette L. F. Girard G. R. Kuo G. Y. (1986). Correlation of the *in vitro* cytotoxic and *in vivo* antitumor activities of gold(I) coordination complexes. J. Med. Chem. 29, 218–223. 10.1021/jm00152a009 3081721

[B21] Murphy M. P. (2009). How mitochondria produce reactive oxygen species. Biochem. J. 417, 1–13. 10.1042/BJ20081386 19061483 PMC2605959

[B22] Murphy M. P. Hartley R. C. (2018). Mitochondria as a therapeutic target for common pathologies. Nat. Rev. Drug Discov. 17, 865–886. 10.1038/nrd.2018.174 30393373

[B23] Pratesi A. Gabbiani C. Michelucci E. Ginanneschi M. Papini A. M. Rubbiani R. (2014). Insights on the mechanism of thioredoxin reductase inhibition by gold N-heterocyclic carbene compounds using the synthetic linear selenocysteine containing C-terminal peptide hTrxR(488-499): an ESI-MS investigation. J. Inorg. Biochem. 136, 161–169. 10.1016/j.jinorgbio.2014.01.009 24524917

[B24] Rajendran V. Jain M. V. (2018). *In vitro* tumorigenic Assay: colony forming Assay for cancer stem cells. Methods Mol. Biol. 1692, 89–95. 10.1007/978-1-4939-7401-6_8 28986889

[B25] Ralph S. J. Low P. Dong L. Lawen A. Neuzil J. (2006). Mitocans: mitochondrial targeted anti-cancer drugs as improved therapies and related patent documents. Recent Pat. Anti-Canc 1, 327–346. 10.2174/157489206778776952 18221044

[B26] Sousa J. S. D'Imprima E. Vonck J. (2018). Mitochondrial respiratory chain complexes. Subcell. Biochem. 87, 167–227. 10.1007/978-981-10-7757-9_7 29464561

[B27] Steers G. J. Chen G. Y. O'Leary B. R. Du J. Van Beek H. Cullen J. J. (2022). Auranofin and pharmacologic ascorbate as radiomodulators in the treatment of pancreatic cancer. Antioxidants-Basel 11, 971. 10.3390/antiox11050971 35624835 PMC9137675

[B28] Supinski G. S. Schroder E. A. Callahan L. A. (2019). Mitochondria and critical illness. Chest 157, 310–322. 10.1016/j.chest.2019.08.2182 31494084 PMC7005375

[B29] Szeliga M. Rola R. (2022). Menadione potentiates auranofin-induced glioblastoma cell death. Int. J. Mol. Sci. 23, 15712. 10.3390/ijms232415712 36555352 PMC9778806

[B30] Tait S. W. Green D. R. (2010). Mitochondria and cell death: outer membrane permeabilization and beyond. Nat. Rev. Mol. Cell Bio. 11, 621–632. 10.1038/nrm2952 20683470

[B31] Tirichen H. Yaigoub H. Xu W. Wu C. Li R. Li Y. (2021). Mitochondrial reactive oxygen species and their contribution in chronic kidney disease progression through oxidative stress. Front. Physiol. 12, 627837. 10.3389/fphys.2021.627837 33967820 PMC8103168

[B32] Tunes L. G. Morato R. E. Garcia A. Schmitz V. Steindel M. Correa-Junior J. D. (2020). Preclinical gold complexes as oral drug candidates to treat leishmaniasis are potent trypanothione reductase inhibitors. Acs Infect. Dis. 6, 1121–1139. 10.1021/acsinfecdis.9b00505 32283915

[B33] Wallis R. S. Ginindza S. Beattie T. Arjun N. Likoti M. Edward V. A. (2021). Adjunctive host-directed therapies for pulmonary tuberculosis: a prospective, open-label, phase 2, randomised controlled trial. Lancet Resp. Med. 9, 897–908. 10.1016/S2213-2600(20)30448-3 PMC8332197 33740465

[B34] Wang H. Gao Z. Liu X. Agarwal P. Zhao S. Conroy D. W. (2018). Targeted production of reactive oxygen species in mitochondria to overcome cancer drug resistance. Nat. Commun. 9, 562. 10.1038/s41467-018-02915-8 29422620 PMC5805731

[B35] Wang J. Li J. Xiao Y. Fu B. Qin Z. (2020). TPP-based mitocans: a potent strategy for anticancer drug design. Rsc Med. Chem. 11, 858–875. 10.1039/c9md00572b 33479681 PMC7489259

[B36] Yeo C. I. Ooi K. K. Tiekink E. (2018). Gold-based medicine: a paradigm shift in anti-cancer therapy? Molecules 23, 1410. 10.3390/molecules23061410 29891764 PMC6100309

