# Supplementary material for: Synthesis and biological evaluation of novel bi-gold mitocans in lung cancer cells
Source: Front Chem. 2023 Dec 11;11:1292115. doi: 10.3389/fchem.2023.1292115 (PMC10750375; doi:10.3389/fchem.2023.1292115)
Supplement: Supplementary file 1 [file Presentation1.pdf]

Supporting Materials

Synthesis and biological evaluation of novel bi-gold mitocans in lung cancer cells

Wenwen Ding, Qingbin Cui, Wenhua Lu, Yongliang Du, Yao Luo, Yumin Hu, Peng Huang, Shijun Wen

State Key Laboratory of Oncology in South China; Collaborative innovation Center for Cancer Medicine; Guangdong Provincial Clinical Research Center for Cancer; Department of Experimental Research, Sun Yat-sen University Cancer Center, Guangzhou 510006, China

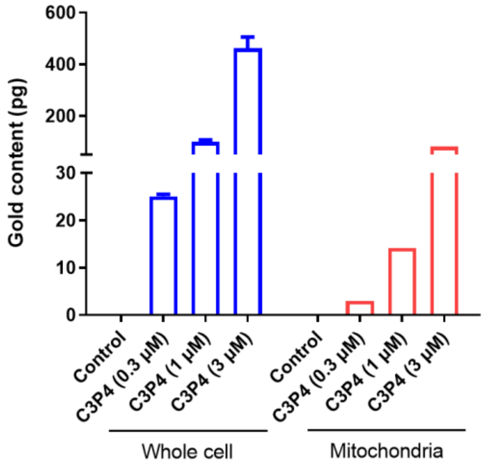

Figure S1. Gold contents in whole cells and isolated mitochondria of each group.

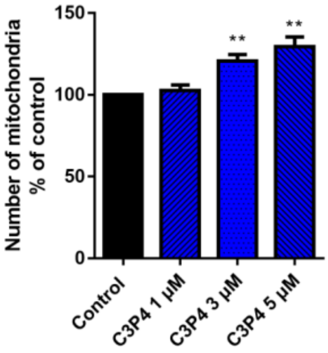

Figure S2. C3P4 at 3 and 5 μM slightly increased mitochondria number. ** $P < 0.01$ *vs* Control.

NMR spectrum

C1P2

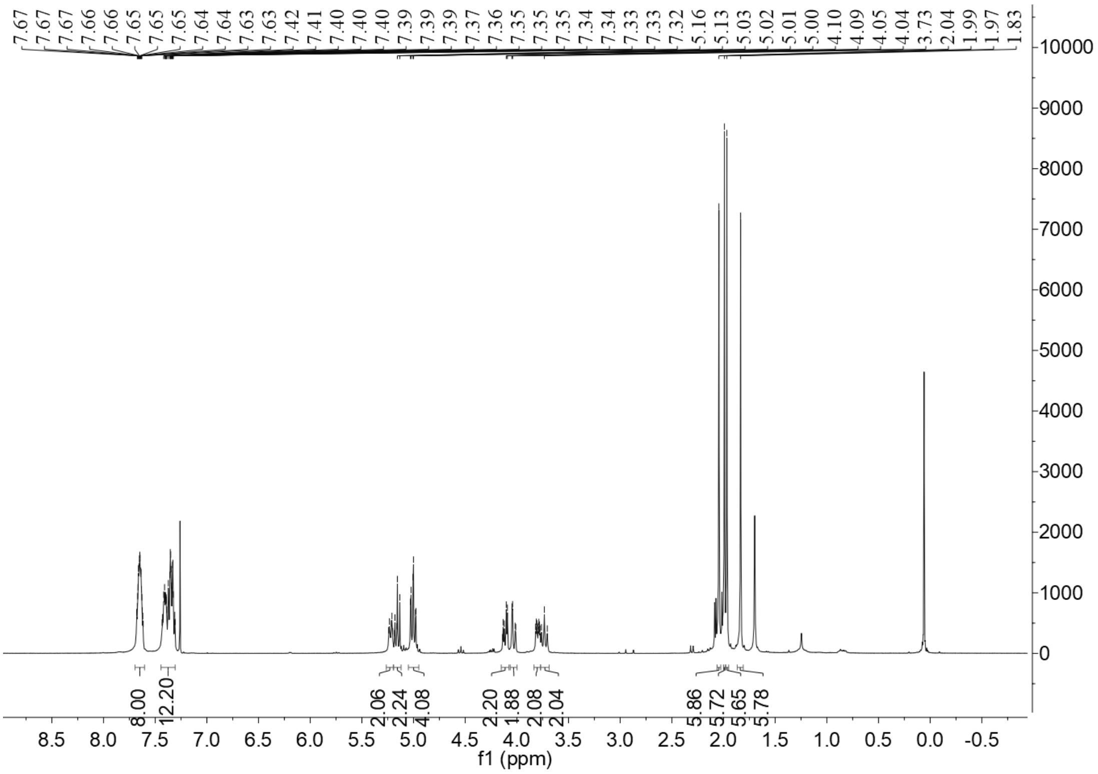

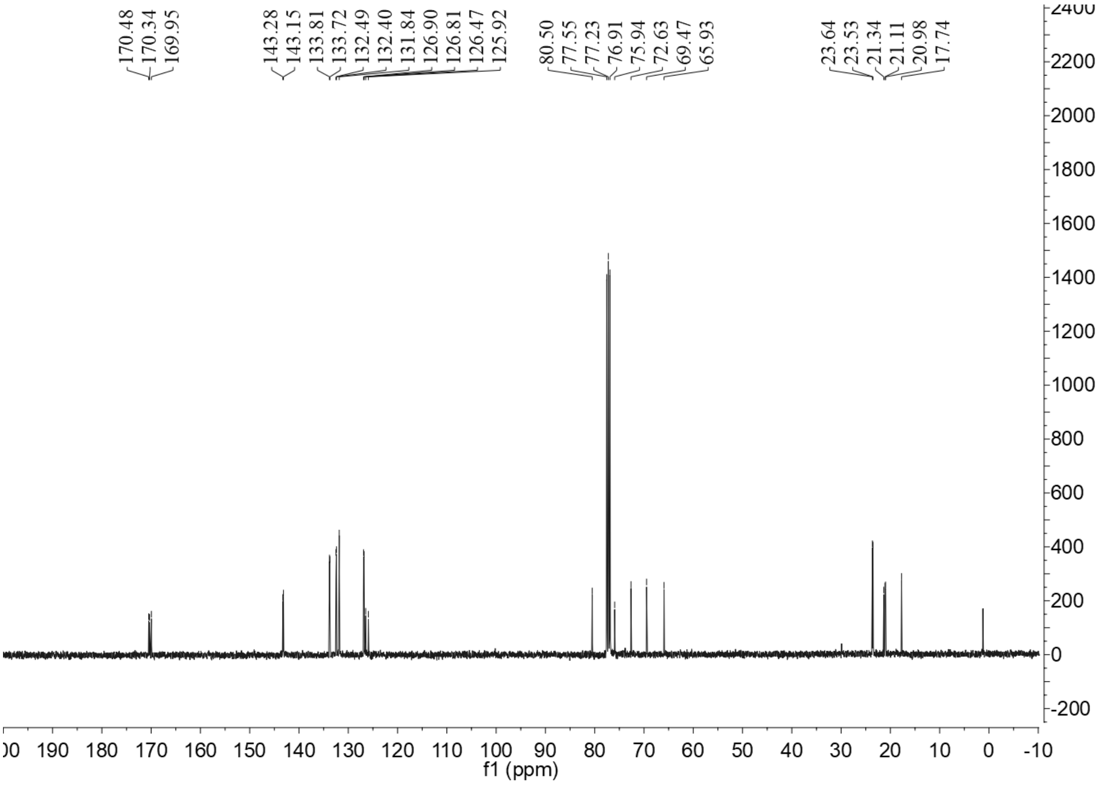

P-NMR

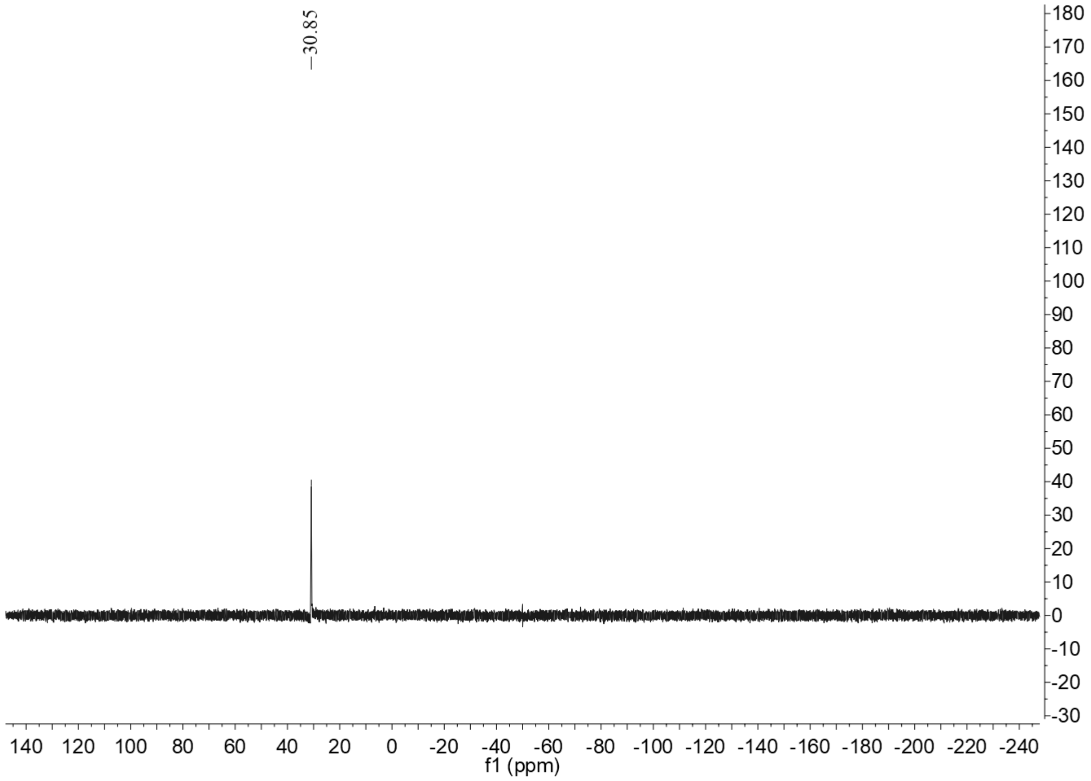

C1P3 (known compound)

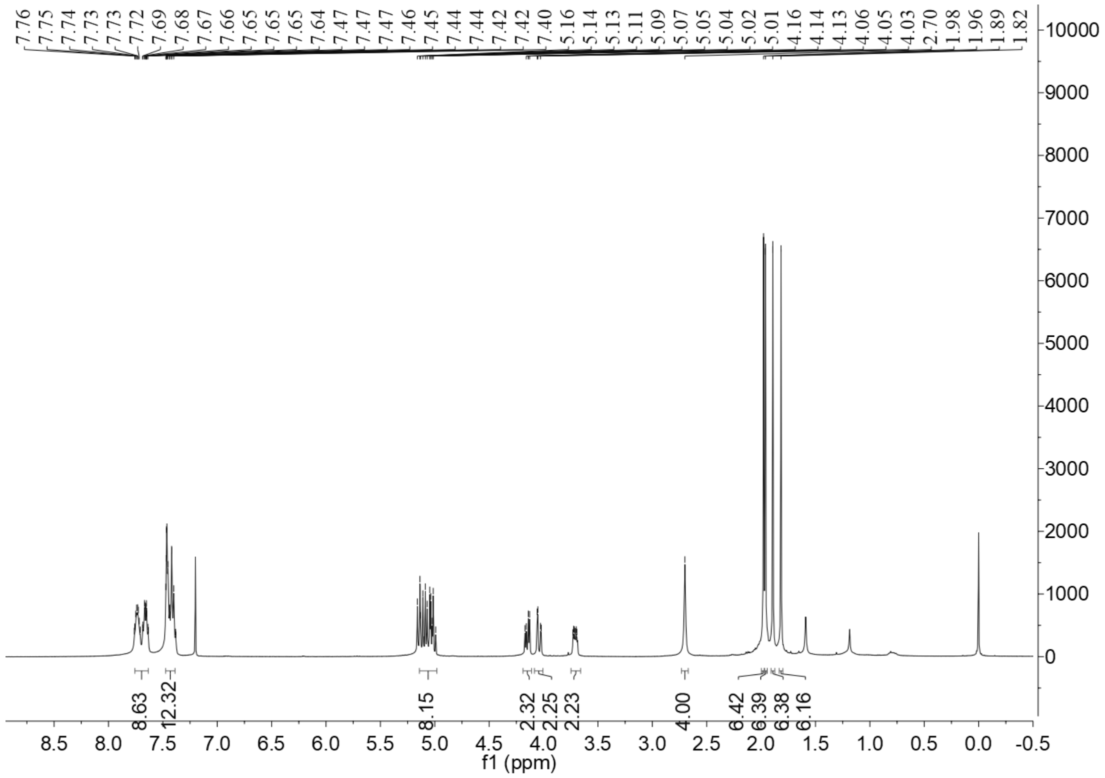

C1P4

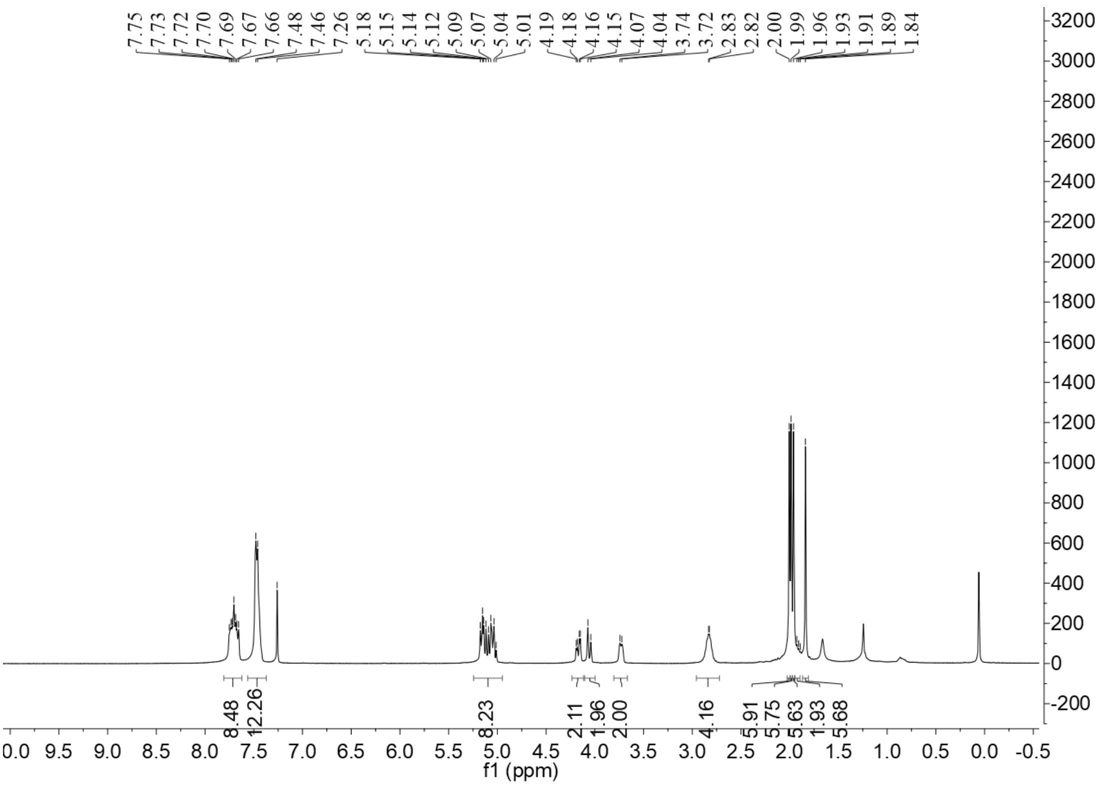
7.75
7.73
7.72
7.70
7.69
7.67
7.66
7.48
7.46
7.26
5.18
5.15
5.14
5.12
5.09
5.07
5.04
5.01
4.19
4.18
4.16
4.15
4.07
4.04
3.74
3.72
2.83
2.82
2.00
1.99
1.96
1.93
1.91
1.89
1.84
8.48
12.26
8.23
2.11
1.96
2.00
4.16
5.91
5.75
5.63
1.93
5.68
f1 (ppm)

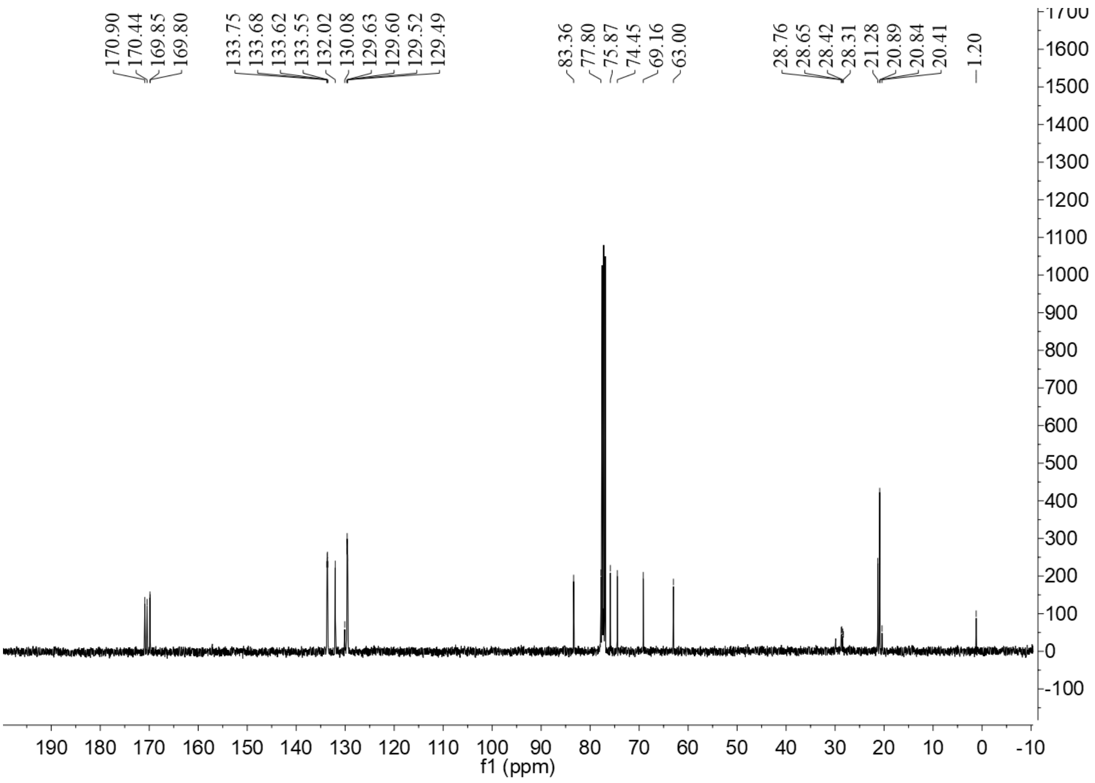
170.90
170.44
169.85
169.80
133.75
133.68
133.62
133.55
132.02
130.08
129.63
129.60
129.52
129.49
83.36
77.80
75.87
74.45
69.16
63.00
28.76
28.65
28.42
28.31
21.28
20.89
20.84
20.41
1.20
f1 (ppm)

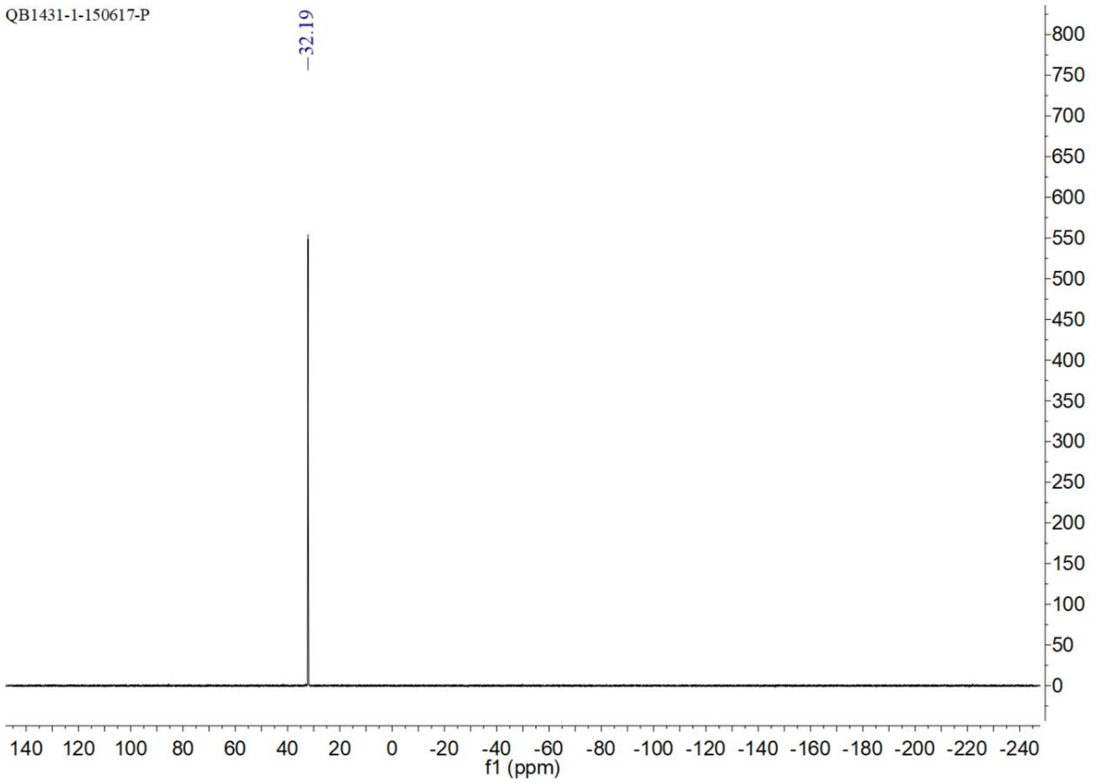

QB1431-1-150617-P
32.19
800
750
700
650
600
550
500
450
400
350
300
250
200
150
100
50
0
140 120 100 80 60 40 20 0 -20 -40 -60 -80 -100 -120 -140 -160 -180 -200 -220 -240
f1 (ppm)

C1P5

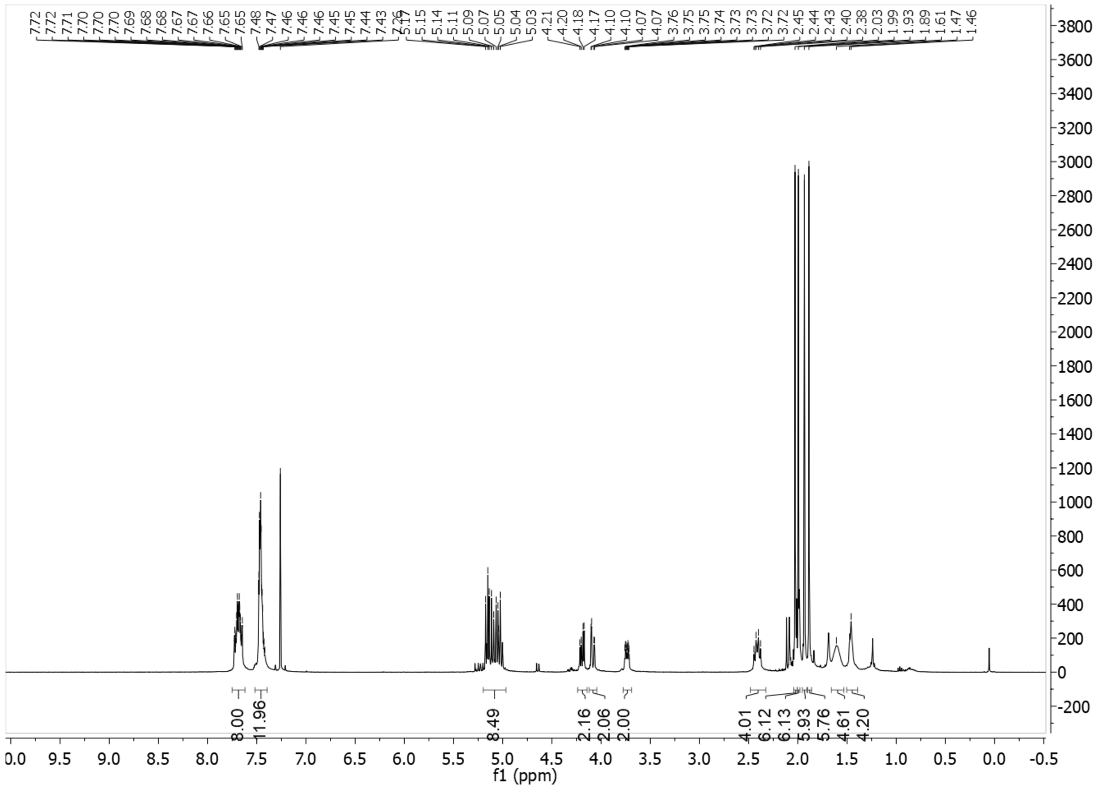

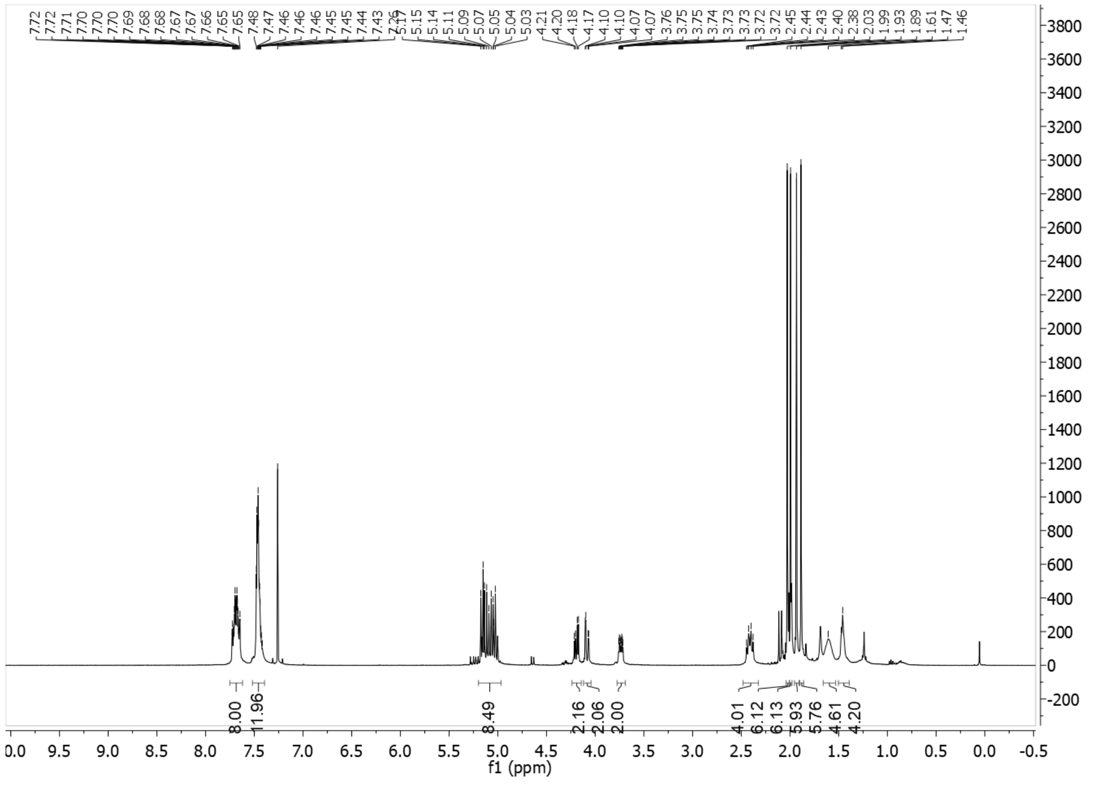

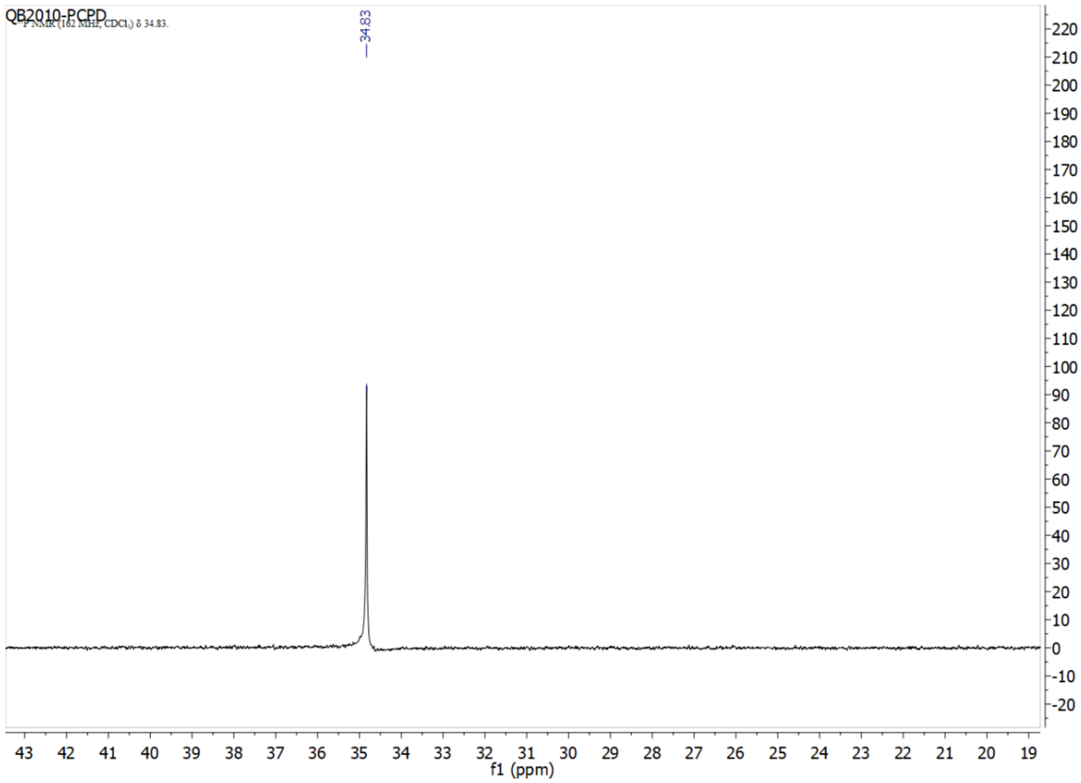
QB2010-PCPD
P NMR (162 MHz, CDCl3) δ 34.83.
34.83
220
210
200
190
180
170
160
150
140
130
120
110
100
90
80
70
60
50
40
30
20
10
0
-10
-20
43 42 41 40 39 38 37 36 35 34 33 32 31 30 29 28 27 26 25 24 23 22 21 20 19
f1 (ppm)

C1P6

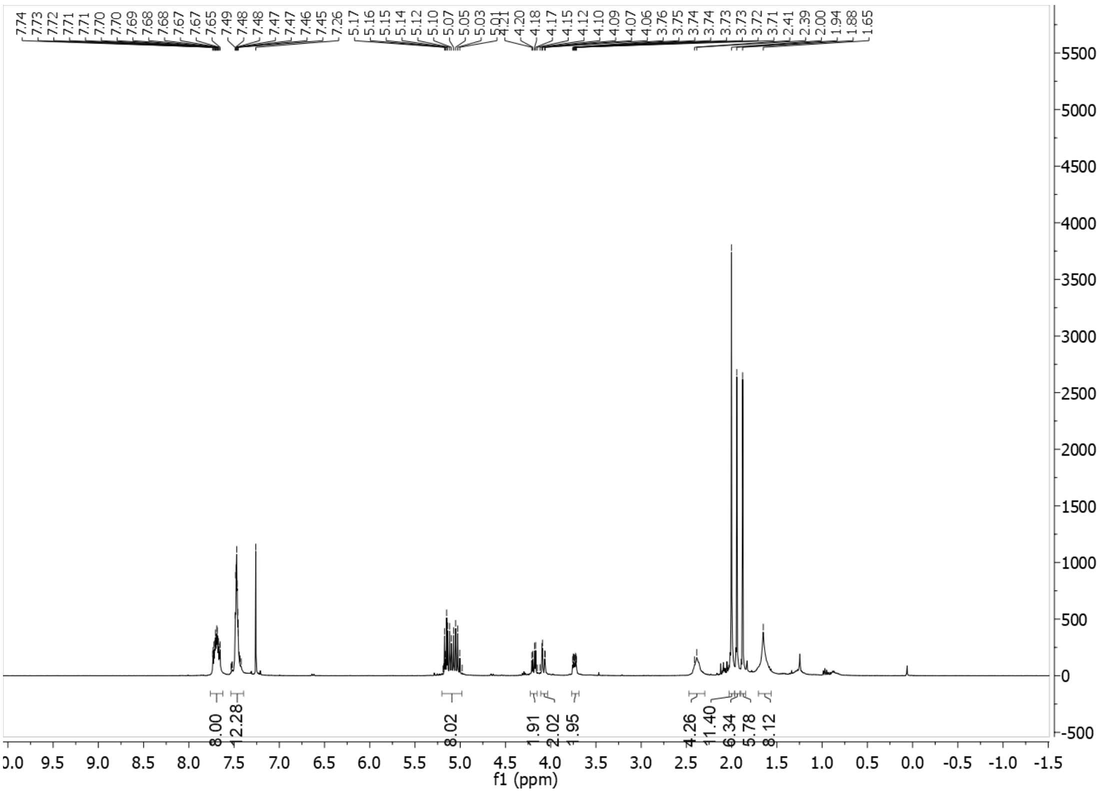
7.74
7.73
7.72
7.71
7.71
7.70
7.70
7.69
7.68
7.68
7.67
7.67
7.65
7.49
7.48
7.47
7.47
7.46
7.45
7.26
5.17
5.16
5.15
5.14
5.12
5.10
5.07
5.05
5.03
5.01
4.21
4.20
4.18
4.17
4.15
4.12
4.10
4.09
4.07
4.06
3.76
3.75
3.74
3.74
3.73
3.73
3.72
3.71
2.41
2.39
2.00
1.94
1.88
1.65
8.00
12.28
8.02
1.91
2.02
1.95
4.26
11.40
6.34
5.78
8.12
f1 (ppm)

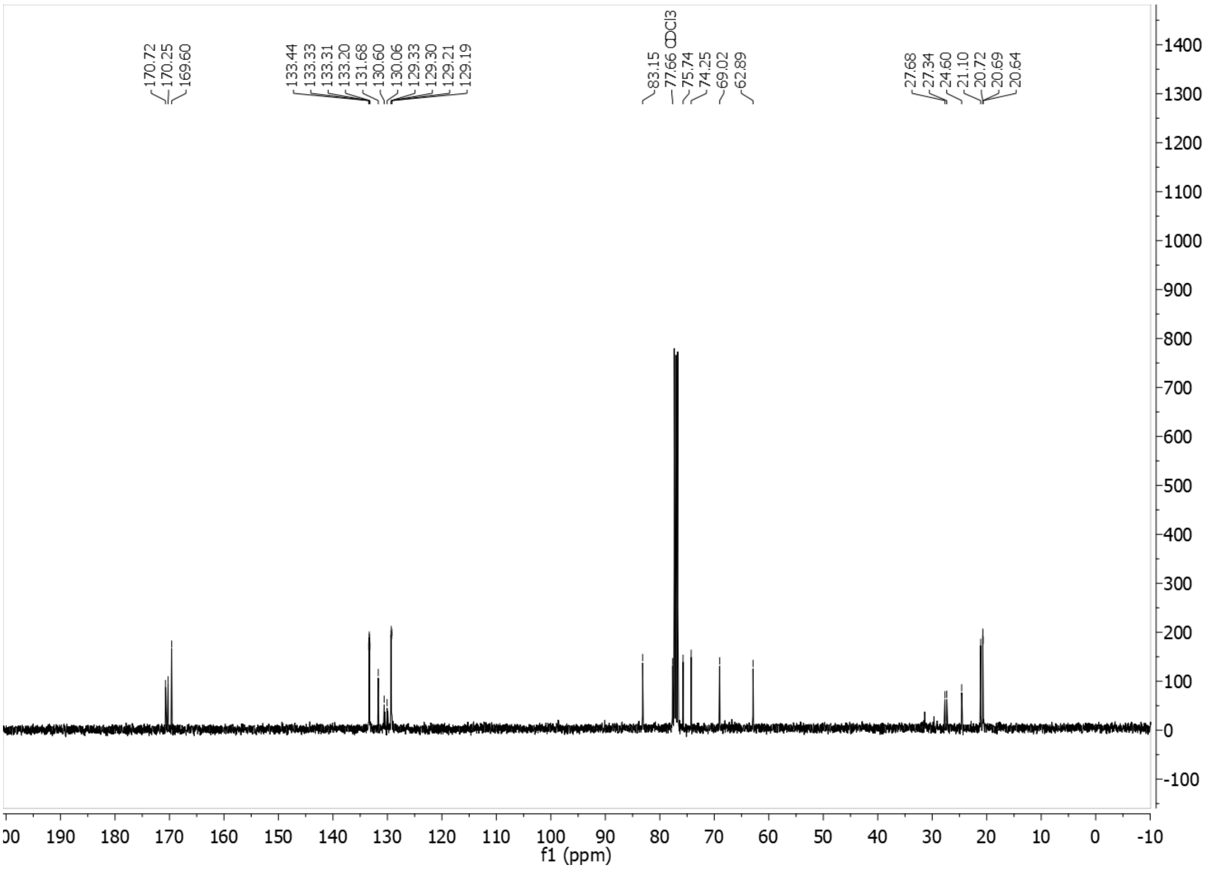
170.72
170.25
169.60
133.44
133.33
133.31
133.20
131.68
130.60
130.06
129.33
129.30
129.21
129.19
83.15
CDCl3
77.66
75.74
74.25
69.02
62.89
27.68
27.34
24.60
21.10
20.72
20.69
20.64
f1 (ppm)

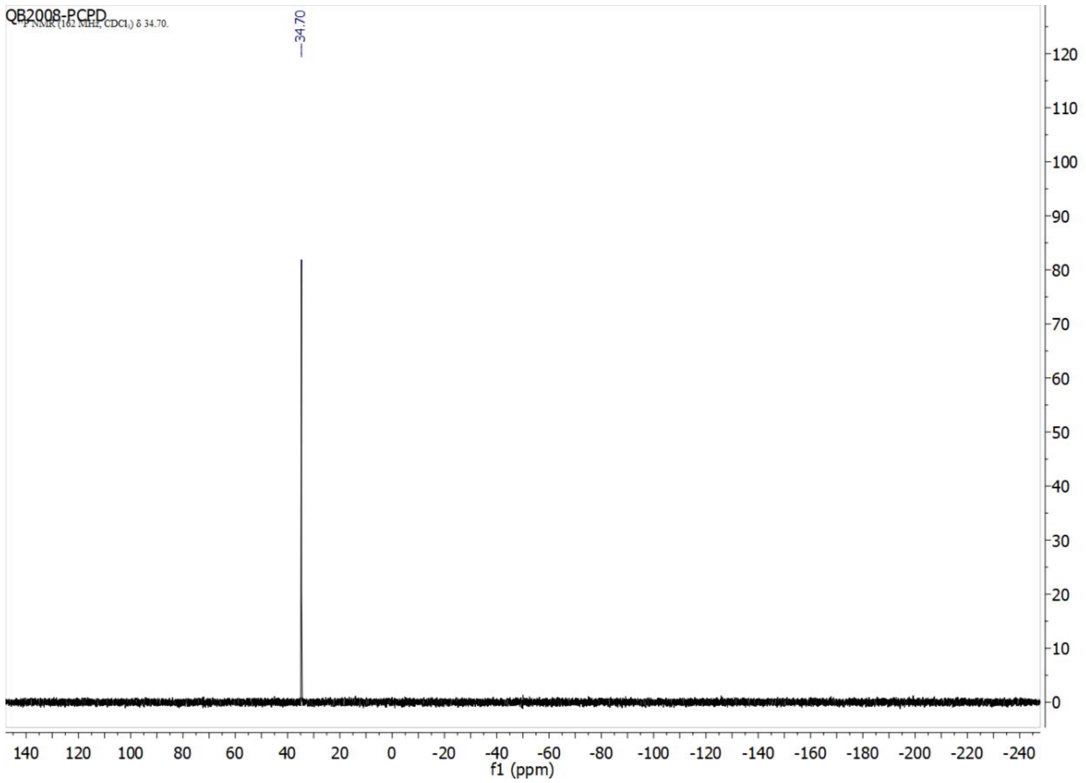
QB2008-PCPD
P NMR (162 MHz, CDCl3) δ 34.70.
34.70
120
110
100
90
80
70
60
50
40
30
20
10
0
140 120 100 80 60 40 20 0 -20 -40 -60 -80 -100 -120 -140 -160 -180 -200 -220 -240
f1 (ppm)

C1P7

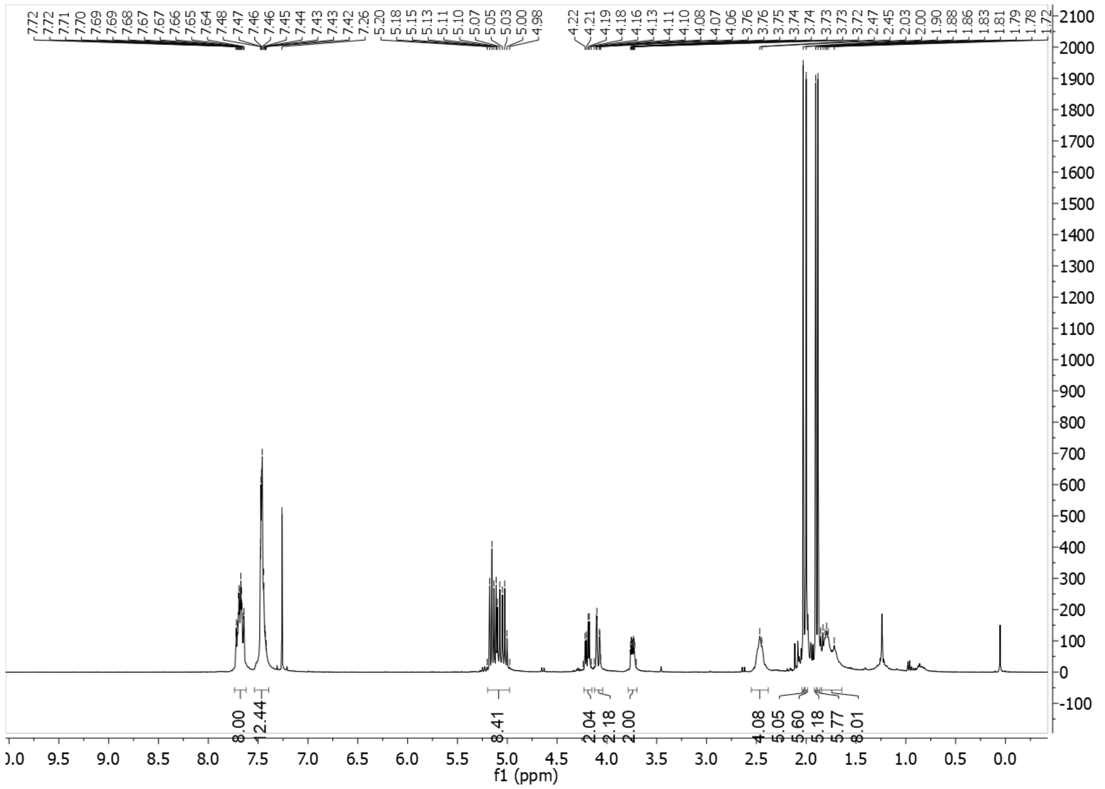

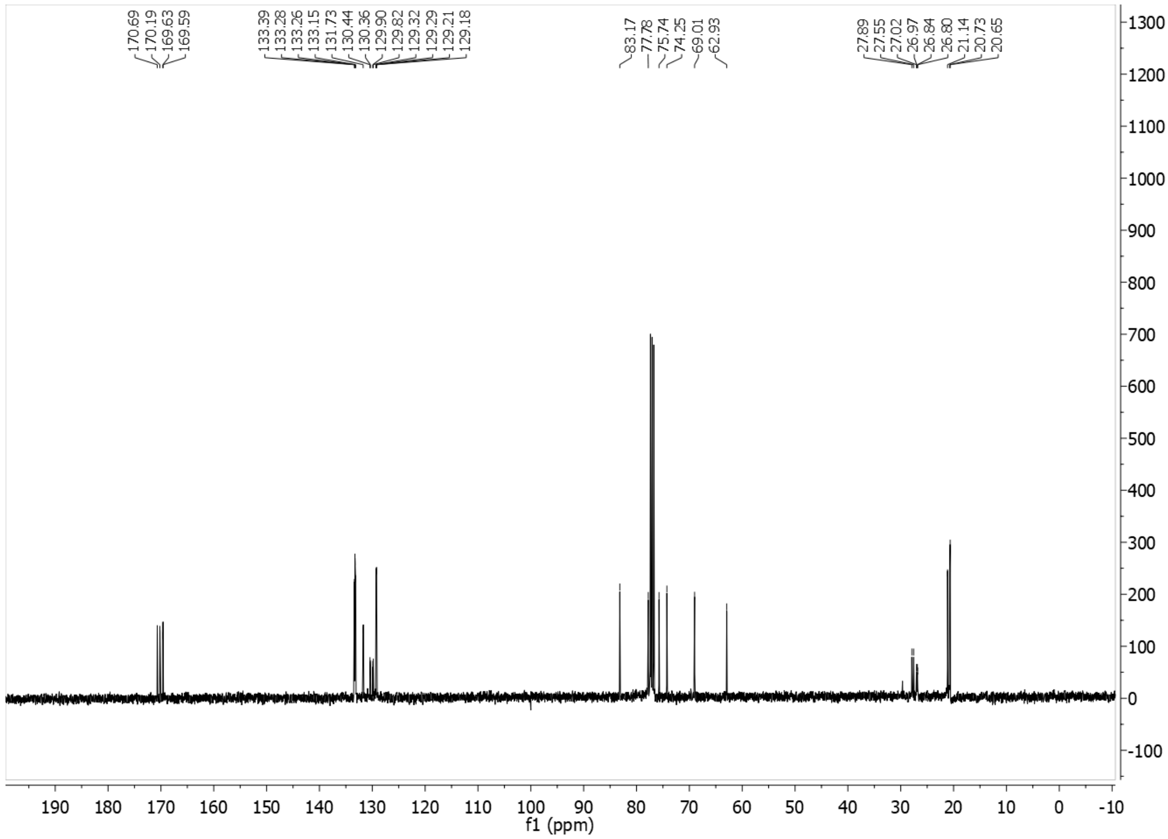

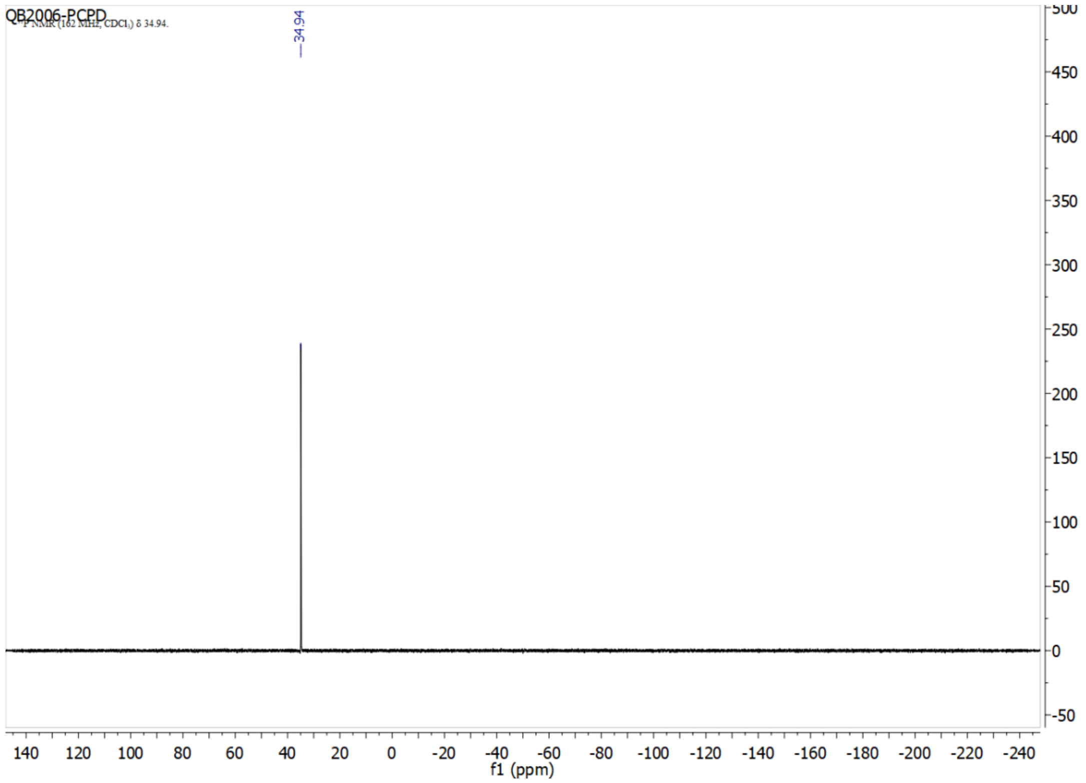
QB2006-PCPD
31P NMR (162 MHz, CDCl3) δ 34.94.
34.94
500
450
400
350
300
250
200
150
100
50
0
-50
140
120
100
80
60
40
20
0
-20
-40
-60
-80
-100
-120
-140
-160
-180
-200
-220
-240
f1 (ppm)

C2P4

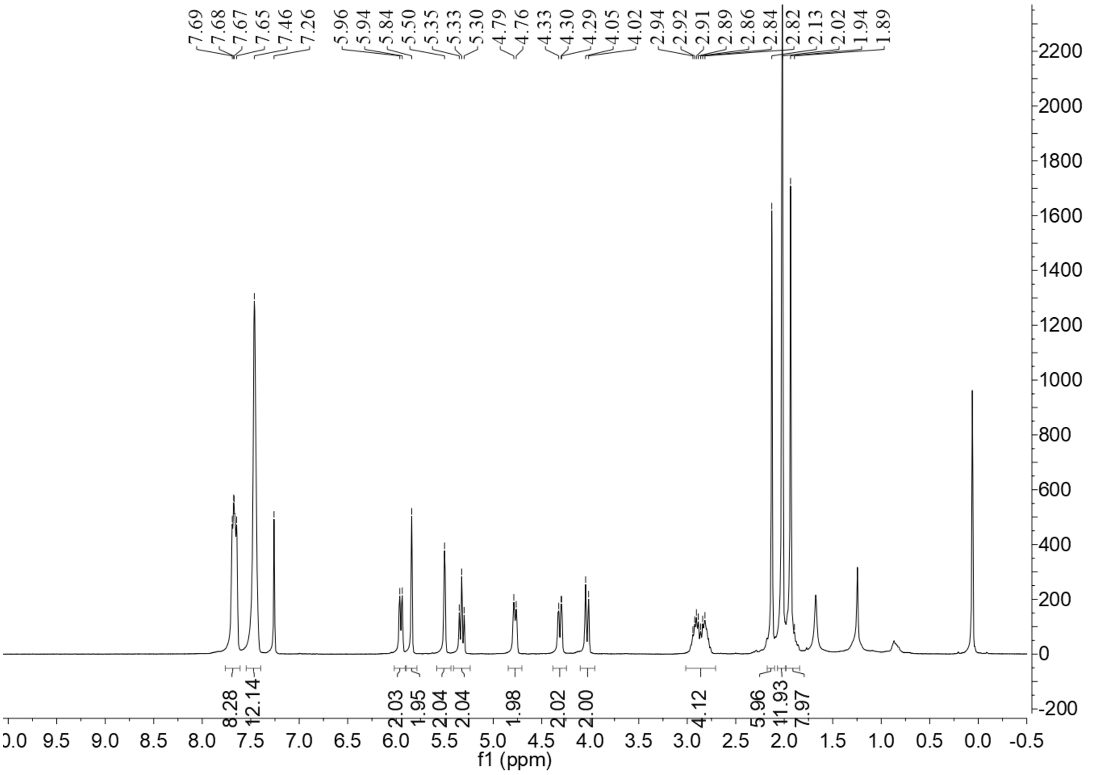

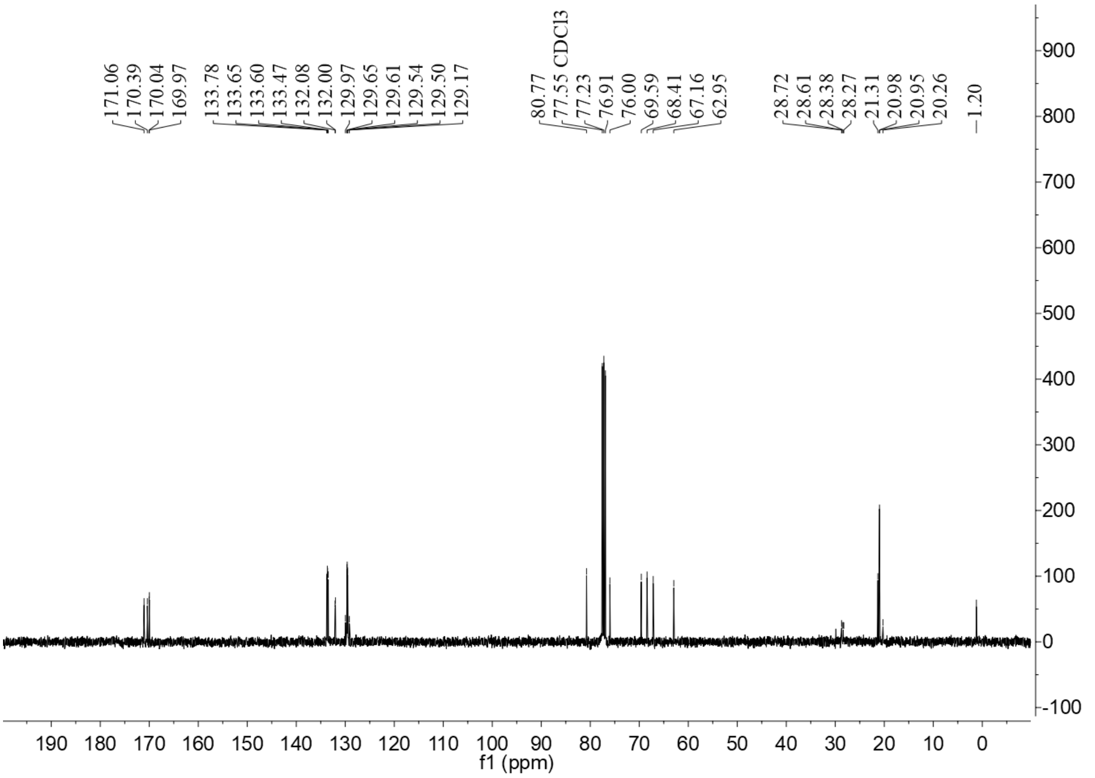

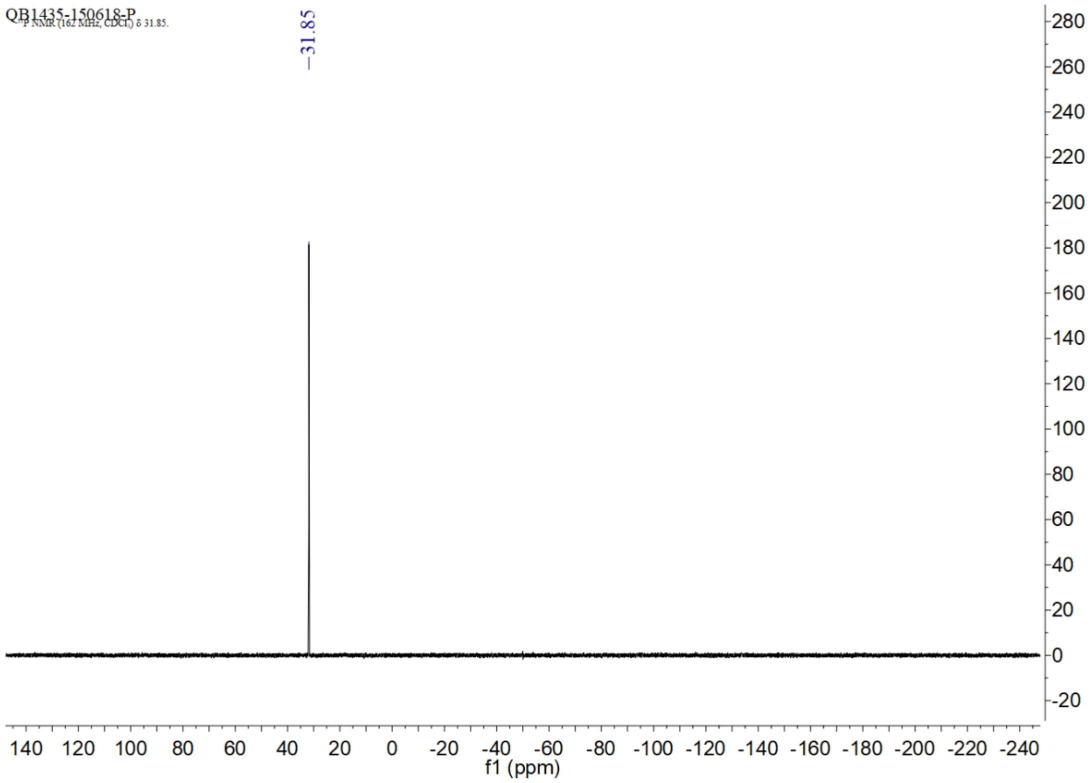

QB1435-150618-P
31P NMR (162 MHz, CDCl3) δ 31.85.
31.85
280
260
240
220
200
180
160
140
120
100
80
60
40
20
0
-20
140 120 100 80 60 40 20 0 -20 -40 -60 -80 -100 -120 -140 -160 -180 -200 -220 -240
f1 (ppm)

C3P4

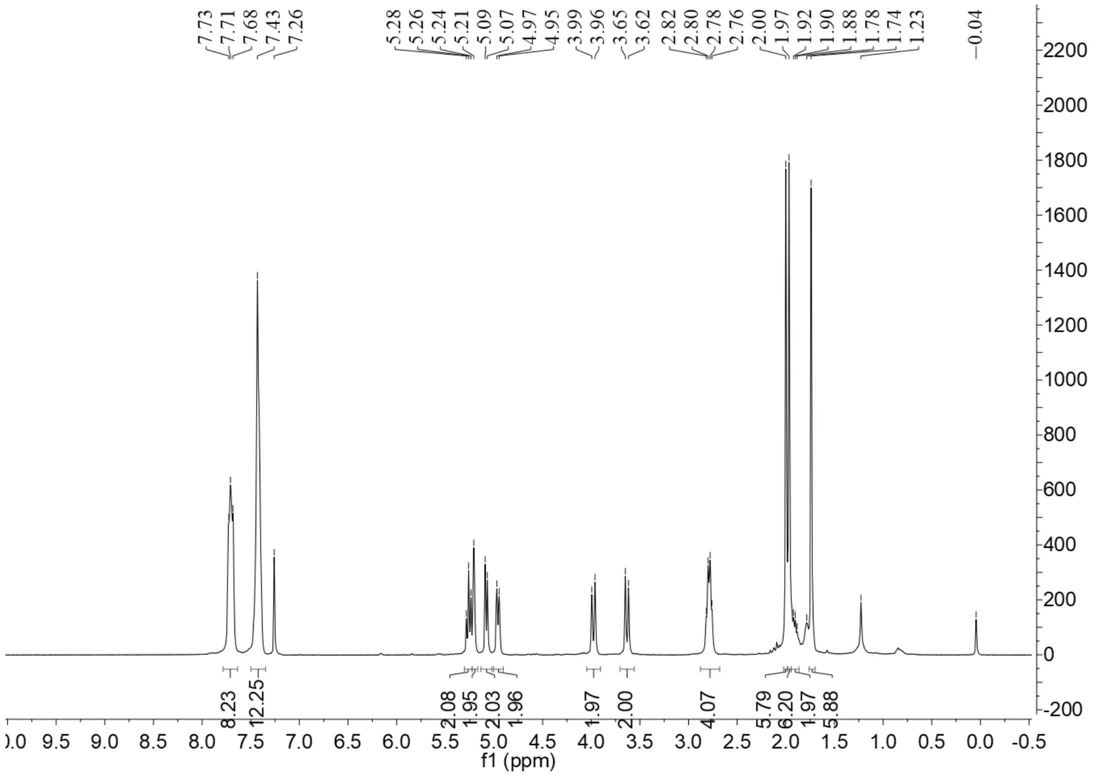
7.73 7.71 7.68 7.43 7.26
5.28 5.26 5.24 5.21 5.09 5.07 4.97 4.95 3.99 3.96 3.65 3.62 2.82 2.80 2.78 2.76 2.00 1.97 1.92 1.90 1.88 1.78 1.74 1.23
0.04
8.23 12.25 2.08 1.95 2.03 1.96 1.97 2.00 4.07 5.79 6.20 1.97 5.88
f1 (ppm)

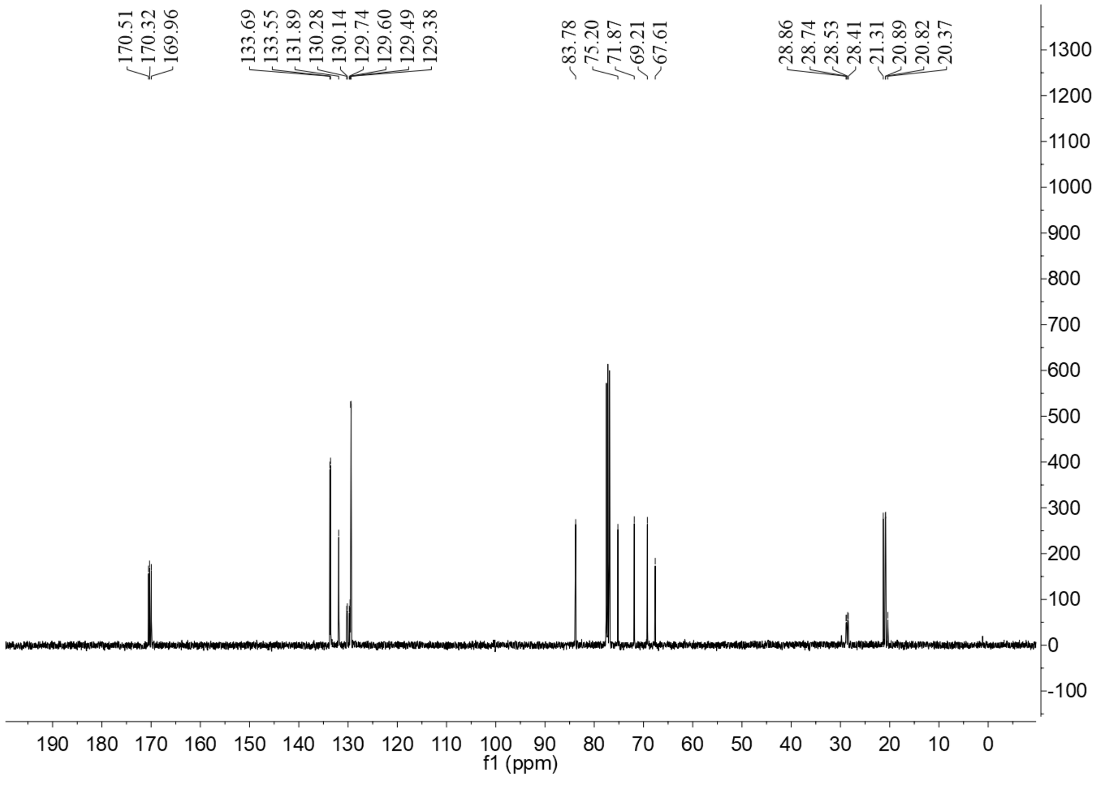
170.51 170.32 169.96
133.69 133.55 131.89 130.28 130.14 129.74 129.60 129.49 129.38
83.78 75.20 71.87 69.21 67.61
28.86 28.74 28.53 28.41 21.31 20.89 20.82 20.37
f1 (ppm)

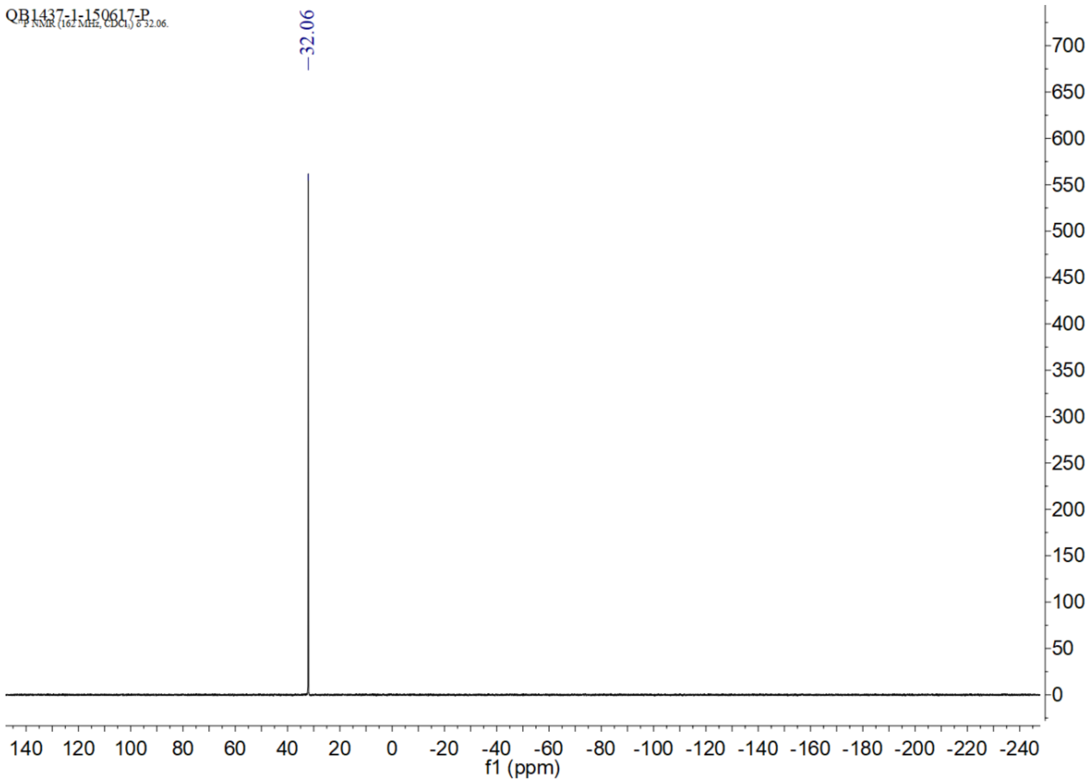
QB1437-1-150617-P
P NMR (162 MHz, CDCl3) δ 32.06.
32.06
700
650
600
550
500
450
400
350
300
250
200
150
100
50
0
140 120 100 80 60 40 20 0 -20 -40 -60 -80 -100 -120 -140 -160 -180 -200 -220 -240
f1 (ppm)

C4P4

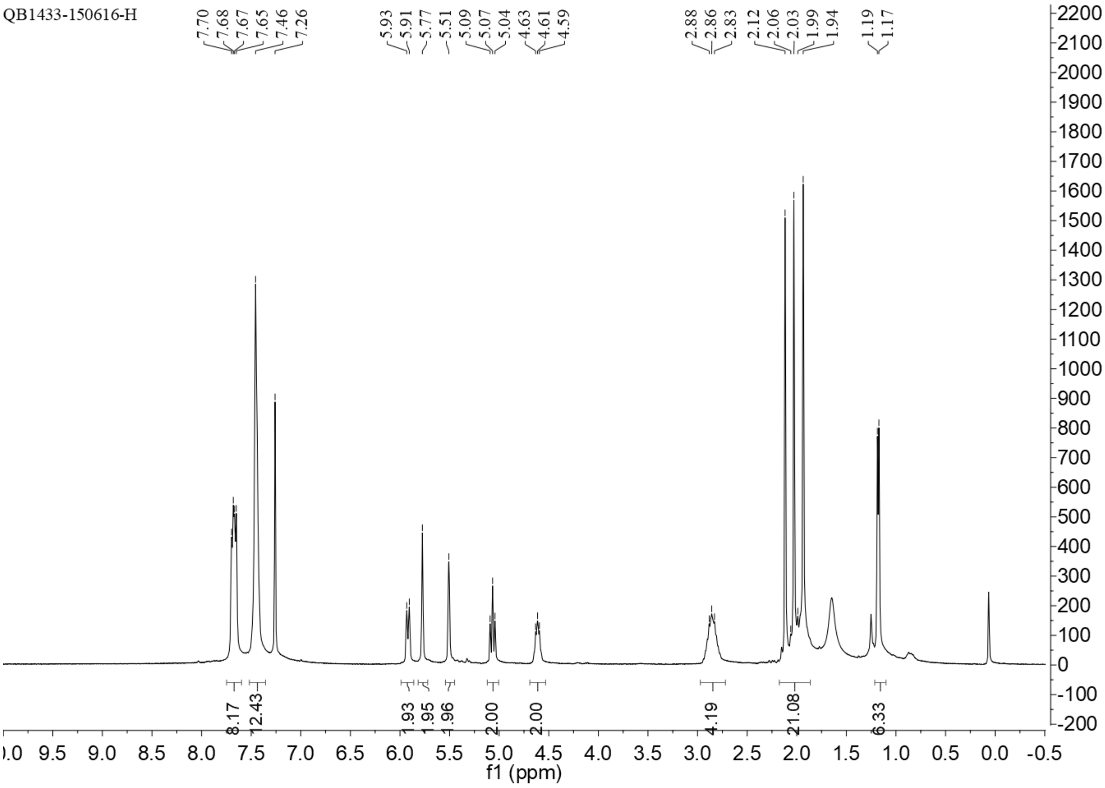
QB1433-150616-H
7.70
7.68
7.67
7.65
7.46
7.26
5.93
5.91
5.77
5.51
5.09
5.07
5.04
4.63
4.61
4.59
2.88
2.86
2.83
2.12
2.06
2.03
1.99
1.94
1.19
1.17
8.17
12.43
1.93
1.95
1.96
2.00
2.00
4.19
21.08
6.33
f1 (ppm)

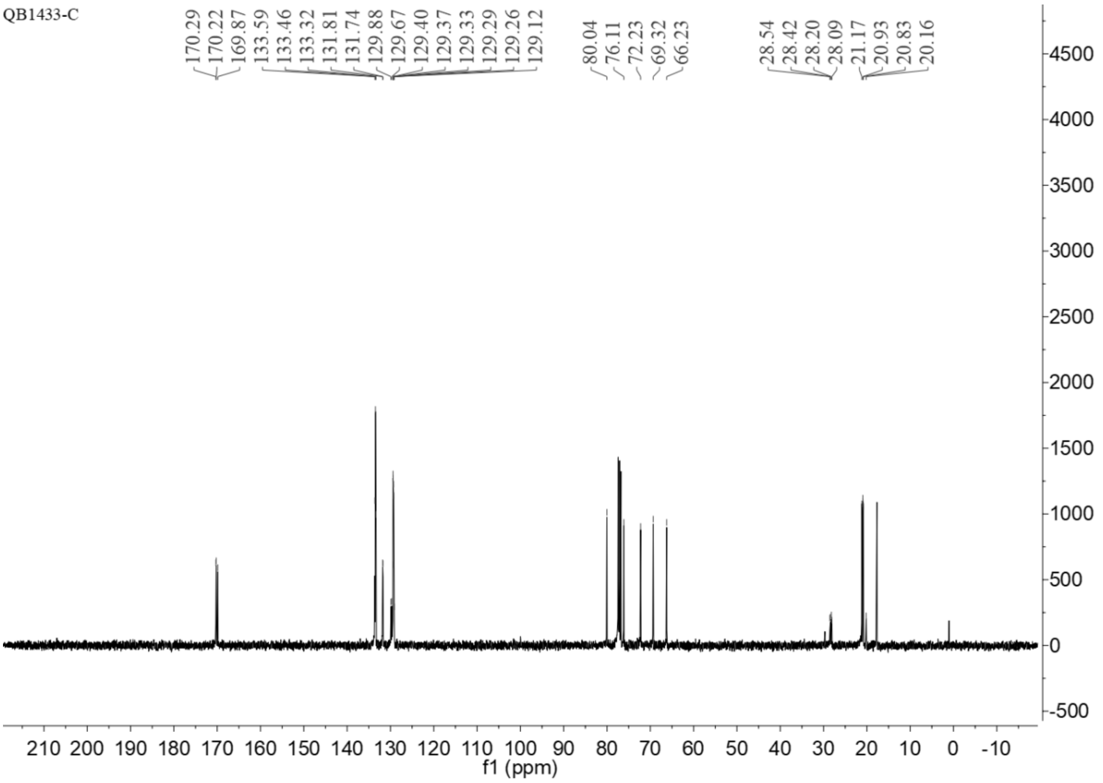
QB1433-C
170.29
170.22
169.87
133.59
133.46
133.32
131.81
131.74
129.88
129.67
129.40
129.37
129.33
129.29
129.26
129.12
80.04
76.11
72.23
69.32
66.23
28.54
28.42
28.20
28.09
21.17
20.93
20.83
20.16
f1 (ppm)

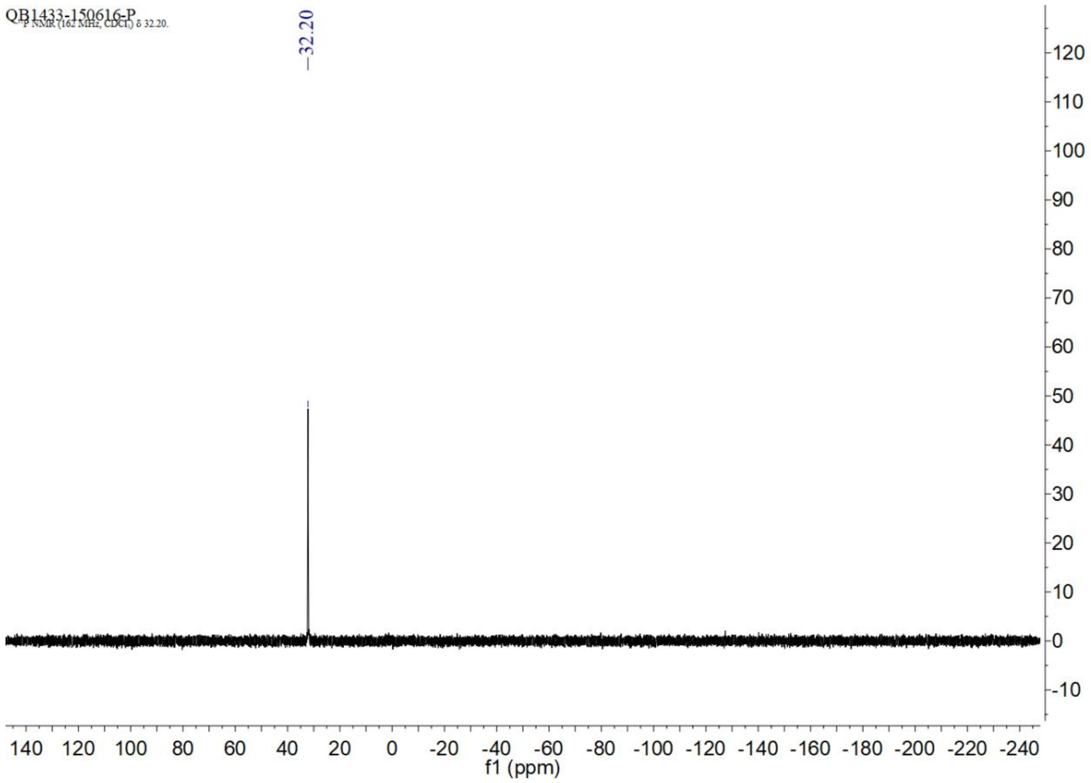
QB1433-150616-P
^{31}P NMR (162 MHz, $CDCl_3$) δ 32.20.
32.20
120
110
100
90
80
70
60
50
40
30
20
10
0
-10
140 120 100 80 60 40 20 0 -20 -40 -60 -80 -100 -120 -140 -160 -180 -200 -220 -240
f1 (ppm)
